# Integrating Molecular Networking and ^1^H NMR Spectroscopy for Isolation of Bioactive Metabolites from the Persian Gulf Sponge *Axinella sinoxea*

**DOI:** 10.3390/md18070366

**Published:** 2020-07-16

**Authors:** Reza Mohsenian Kouchaksaraee, Mahdi Moridi Farimani, Fengjie Li, Melika Nazemi, Deniz Tasdemir

**Affiliations:** 1 Department of Phytochemistry, Medicinal Plants and Drugs Research Institute, Shahid Beheshti University, G. C., Evin, 1983969411 Tehran, Iran; r_mohsenian@sbu.ac.ir; 2 GEOMAR Centre for Marine Biotechnology (GEOMAR-Biotech), Research Unit Marine Natural Products Chemistry, GEOMAR Helmholtz Centre for Ocean Research Kiel, Am Kiel-Kanal 44, 24106 Kiel, Germany; fli@geomar.de; 3 Persian Gulf and Oman Sea Ecological Center, Iranian Fisheries Science Research Institute, Agricultural Research, Education and Extension Organization (AREEO), 7916793165 Bandar Abbas, Iran; melikanazemi@yahoo.com; 4 Faculty of Mathematics and Natural Sciences, Kiel University, Christian-Albrechts-Platz 4, 24118 Kiel, Germany

**Keywords:** marine sponge, *Axinella sinoxea*, Persian Gulf, molecular networking, ^1^H NMR spectroscopy, diketopiperazine, steroid, monoterpene

## Abstract

The geographic position, highly fluctuating sea temperatures and hypersalinity make Persian Gulf an extreme environment. Although this unique environment has high biodiversity dominated by invertebrates, its potential in marine biodiscovery has largely remained untapped. Herein, we aimed at a detailed analysis of the metabolome and bioactivity profiles of the marine sponge *Axinella sinoxea* collected from the northeast coast of the Persian Gulf in Iran. The crude extract and its Kupchan subextracts were tested in multiple in-house bioassays, and the crude extract and its CHCl_3_-soluble portion showed in vitro antibacterial activity against Methicillin-resistant *Staphylococcus aureus* (MRSA) and *Enterococcus faecium* (Efm). A molecular networking (MN)-based dereplication strategy by UPLC-MS/MS revealed the presence of phospholipids and steroids, while ^1^H NMR spectroscopy indicated the presence of additional metabolites, such as diketopiperazines (DKPs). Integrated MN and ^1^H NMR analyses on both the crude and CHCl_3_ extracts combined with an antibacterial activity-guided isolation approach afforded eight metabolites: a new diketopiperazine, (-)-cyclo(*L*-*trans*-Hyp-*L*-Ile) (**8**); a known diketopiperazine, cyclo(*L*-*trans*-Hyp-*L*-Phe) (**7**); two known phospholipids, 1-*O*-hexadecyl-*sn*-glycero-3-phosphocholine (**1**) and 1-*O*-octadecanoyl-*sn*-glycero-3-phosphocholine (**2**); two known steroids, 3β-hydroxycholest-5-ene-7,24-dione (**3**) and (22*E*)-3β-hydroxycholesta-5,22-diene-7,24-dione (**4**); two known monoterpenes, loliolide (**5**) and 5-*epi*-loliolide (**6**). The chemical structures of the isolates were elucidated by a combination of NMR spectroscopy, HRMS and [α]_D_ analyses. All compounds were tested against MRSA and Efm, and compound **3** showed moderate antibacterial activity against MRSA (IC_50_ value 70 μg/mL). This is the first study that has dealt with chemical and bioactivity profiling of *A.* *sinoxea* leading to isolation and characterization of pure sponge metabolites.

## 1. Introduction

Iranian coastline totals 1700 km and borders the Persian Gulf and Gulf of Oman (GO) in the northwest Indian Ocean. The Gulf of Oman is an exposed and deep component of the Arabian Sea in the northwest Indian Ocean, while the Persian Gulf is a warm, shallow and enclosed sea separated from the Arabian Sea by the GO. The Persian Gulf is an extension of the Indian Ocean and is connected to the GO by the Strait of Hormuz. The average depth of the Persian Gulf is around 35 m, and the maximum depth can reach 100 m. Due to extreme aridity and high summer temperatures up to 48 °C, water surface temperature fluctuates between 12 to 35 °C [1,2]. The evaporation of the Persian Gulf exceeds freshwater input tenfold (commonly measured at 40–50 ppt and up to 70 ppt in shallow, enclosed bays) leading to high salinity. These natural pressures, as well as high anthropogenic stress due to industrialization and urbanization, render the Persian Gulf an extreme environment [2]. Nevertheless, the fauna of the Persian Gulf is still highly diverse and dominated by the sponge and coral communities. A recent study has investigated the sponge diversity of the Persian Gulf collected from Iran [3]. However, the chemistry and biomedical potential of sponges inhabiting the Persian Gulf have remained largely untapped so far.

The sponges of the family Axinellidae are common in many oceans, such as the Indian and Pacific Oceans, including the Persian Gulf. *Axinella* is a large sponge genus comprising 183 species [4]. The members of this genus are prolific sources of a wide array of natural product classes, such as steroids, sesquiterpenoids, diterpenoids, alkaloids, polyethers and cyclic peptides (including DKPs) [5,6,7,8,9,10]. The extracts, as well as secondary metabolites isolated from *Axinella* species, have been reported to exhibit diverse biological activities, with antimicrobial [7,8] and anticancer activities [11,12,13] being probably the most common ones. In the Persian Gulf, the genus *Axinella* is represented by a single species, *A. sinoxea* [13]. As part of our project aiming at in-depth metabolome and bioactivity profiling of Persian Gulf sponges, we have selected *A. sinoxea* that Alvarez and Hooper, 2009 collected from Larak Island (Iran) for comprehensive chemical studies. The crude MeOH extract was screened for biological activities, namely antimicrobial (against the bacterial ESKAPE panel and two fungi) and anticancer (against six human cancer cell lines and one non-cancerous cell line). The crude MeOH extract of the sponge and its CHCl_3_-soluble portion showed considerable antibacterial activity. Hence, we applied an integrated metabolomics approach using both HRMS/MS-based molecular networking and ^1^H NMR spectroscopy to map chemical diversity of the sponge and combined this strategy with a bioactivity-guided isolation procedure for targeted isolation of bioactive and chemically interesting metabolites. This report outlines the chemical and bioactivity profiling of the sponge extracts followed by purification, characterization and bioactivity assessment of compounds **1**–**8** isolated from *A. sinoxea*.

## 2. Results

### 2.1. Bioactivity Profiling

**Figure 1:**
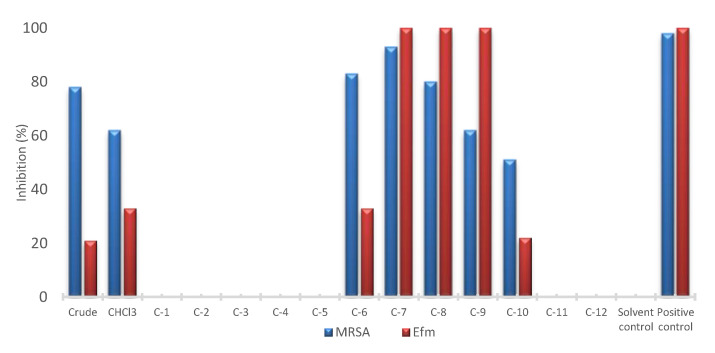
Comparative in vitro activity (% inhibition at 100 µg/mL) of the crude MeOH extract, CHCl_3_ subextract and its C18 fractions against *Staphylococcus aureus* (MRSA) and *Enterococcus faecium* (Efm). Positive controls: chloramphenicol (MRSA) and ampicillin (Efm). Solvent control: 0.5% dimethyl sulfoxide (DMSO).

The sponge material was extracted with MeOH and screened for its in vitro biological activities, including antimicrobial activity against the bacterial ESKAPE panel, i.e., *Enterococcus faecium* (Efm), methillicin-resistant *Staphylococcus aureus* (MRSA), *Klebsiella pneumoniae*, *Acinetobacter baumannii*, *Pseudomonas aeruginosa* and *Escherichia coli*; for antifungal activity against *Candida albicans* and *Cryprococcus neoformans*, and for anticancer activity against six cancer cell lines including lung carcinoma cell line A549, colorectal adenocarcinoma cell line HT29, malignant melanoma cell line A375, liver cancer cell line Hep G2, colon cancer cell line HCT116, human breast cancer line MDA-MB231, plus the non-cancerous human keratinocyte cell line HaCaT. The only high activity demonstrated by the crude MeOH extract was against MRSA (78% inhibition at 100 μg/mL) (Figure 1). The crude extract was partitioned between *n*-hexane, chloroform and aqueous methanol through a modified Kupchan method [14]. The CHCl_3_ subextract retained the anti-MRSA activity and displayed a low antibacterial potency against Efm (62% and 33% inhibition at 100 μg/mL, respectively). This subextract was fractionated by C18 flash chromatography to yield 12 fractions (C1–C12). Repeated HPLC separations of the most active fractions C6–C10 (Figure 1) monitored by anti-MRSA and anti-Efm activities yielded compounds **1**–**6**, while compounds **7** and **8** were isolated from the inactive fraction C1 monitored by the ^1^H NMR profiling.

### 2.2. Molecular Networking-Based Dereplication and ^1^H NMR Profiling

**Figure 2:**
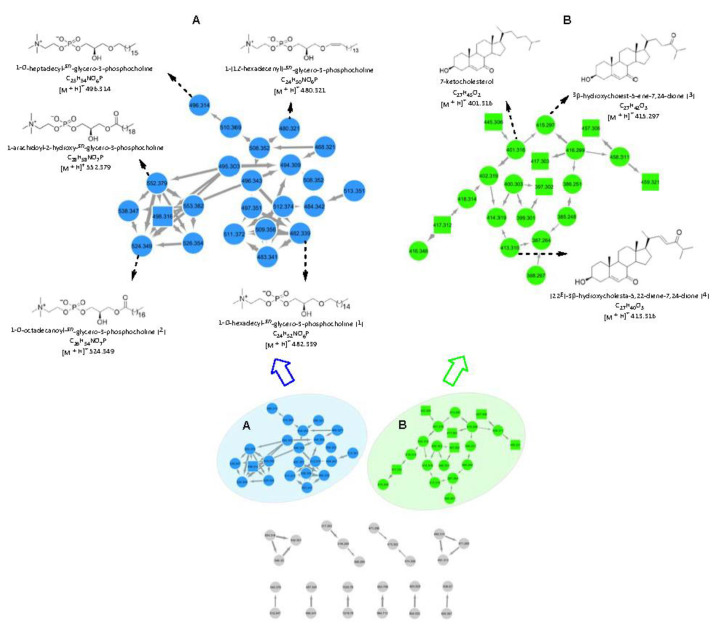
Global molecular network (MN) of the crude extract of *A. sinoxea*. (**A**) Phospholipid cluster (highlighted in blue). (**B**) Steroid cluster (highlighted in green). Numbers within the nodes indicate parent ions, and edge thickness represents the cosine similarity between nodes. Square nodes represent putative new compounds.

Both molecular networking (MN)-based automated and manual dereplication strategies by tandem UPLC-QToF-MS in positive-ion mode were applied for comprehensive metabolite profiling of the crude extract. For data examination purposes, the Global Natural Products Social Molecular Networking (GNPS; www.gnps.ucsd.edu) platform was used [15]. The outcoming MN was visualized via the free software Cytoscape 3.7.1, wherein 12 sub-networks (at least two nodes per cluster) composed of 68 nodes were observed (Figure 2). Not all these nodes correspond to a single molecule as some represent isotopes or adducts. As shown in Figure 2, automated dereplication using the GNPS platform assisted the annotation of two large molecular clusters belonging to phosphatidylcholine (A, blue nodes) and cholesterol (B, green nodes).

Cluster A contained 23 nodes, corresponding to 13 molecules (Figure 2A). Automated dereplication on the GNPS platform allowed the annotation of five known phospholipids, i.e., 1-*O*-heptadecyl-*sn*-glycero-3-phosphocholine (*m/z* 496.314 [M + H]^+^), 1-(1*Z*-hexadecenyl)-*sn*-glycero-3-phosphocholine (*m/z* 480.321 [M + H]^+^), 1-*O*-octadecanoyl-*sn*-glycero-3-phosphocholine (*m/z* 524.349 [M + H]^+^) (**2**), 1-arachidoyl-2-hydroxy-*sn*-glycero-3-phosphocholine (*m/z* 552.379 [M + H]^+^) and 1-*O*-hexadecyl-*sn*-glycero-3-phosphocholine (*m/z* 482.339 [M + H]^+^) (**1**) [16,17,18]. Another node at *m/z* 498.316 (square node, Figure 2A), connected to the known phosphocholine subnetwork with thick edges, was annotated as a putative new phosphocholine analogue as its molecular formula did not match any known compound from this chemical family in a comprehensive database search. Given the elemental composition analysis, biological source and fragmentation patterns, manual dereplication allowed the annotation of all other nodes in cluster A as known phospholipids with varied chain length and unsaturation (Table S1).

Cluster B was annotated as a cholesterol type steroid family based on the elemental composition analysis and MS^2^ fragmentation patterns (Figure 2B). Of the 21 nodes observed in cluster B, one was automatically annotated as the known compound 7-ketocholesterol (*m/z* 401.316 [M + H]^+^) [19] via the GNPS platform. Six nodes at *m/z* 445.306, 457.306, 459.321, 417.312, 417.303 and 397.302 (square nodes, Figure 2B, all *m/z* values are [M + H]^+^), were annotated as putative new cholesterol derivatives based on their similar fragmentation patterns to that of 7-ketocholesterol and the absence of formula matches to any known cholesterols in multiple databases. All remaining nodes in B were dereplicated as known cholesterol analogues manually on the basis of their fragmentation patterns, elemental composition analysis and biological source, e.g., 3β-hydroxycholest-5-ene-7,24-dione (*m/z* 415.297 [M + H]^+^) (**3**) and (22*E*)-3β-hydroxycholesta-5,22-diene-7,24-dione (*m/z* 413.316 [M + H]^+^) (**4**) (Figure 2B) [20]. The supplementary Table S1 demonstrates the detailed MS data of the other annotated steroids.

Apart from the networks A and B, ten small clusters with two or three nodes were visible in the global MN of the crude MeOH extract (Figure 2, the grey networks). They remained unannotated, by both automated and manual dereplication efforts, indicating them to represent putatively new chemical families and compounds.

The CHCl_3_ subextract was also profiled by tandem MS, resulting in altogether 11 clusters (at least two nodes per cluster) that were composed of 72 nodes (Figure S1). Similar to the crude MeOH extract, two big clusters (A1 and B1) dominated the global MN profile. The clusters A1 and B1 were also annotated as phospholipids and cholesterols, respectively, via automated dereplication on the GNPS platform. Cluster A1 contained 29 nodes, generated by 13 molecules, and cluster B1 consisted of 22 nodes, corresponding to 12 molecules. In-depth manual dereplication revealed the composition of both clusters A1 and B1 to the same as that of clusters A and B observed in the global MN of the crude MeOH extract of *A. sinoxea* (Table S1). Remaining small clusters in the global MN (Figure S1) could not be annotated to any known chemical classes, either by automated or manual dereplication methods.

Since MS-based profiling yielded only limited information on the chemical inventory of *A. sinoxea,* we decided to investigate the crude extract and the CHCl_3_ subextract by ^1^H NMR spectroscopy. The ^1^H NMR spectrum of the crude MeOH extract (Figure S2) contained resonances typical for aliphatic lipids (approx. *δ*_H_ 1.0–2.5), methyl signals due to steroids (*δ*_H_ 0.7–1.2), double bonds of unsaturated lipids/fatty acids (*δ*_H_ 5.3) as well as complex aromatic signals (*δ*_H_ 7.1–7.2) and heteroatom-bearing proton signals (*δ*_H_ 3.2–4.5). The ^1^H NMR spectrum of the CHCl_3_ subextract was also highly similar (Figure S3). It contained predominant signals in the aromatic region (*δ*_H_ 7.0–7.2) and several sharp heteroatom-neighboring protons (*δ*_H_ 3.2–3.5 and 4.0–4.5) that were reminiscent of α-methine protons of amino acids and DKPs [21,22]. The information gained from the ^1^H NMR spectrum of the crude and the CHCl_3_ extracts indicated that metabolome of the sponge was richer and UPLC-MS/MS-based profiling did not reflect the full chemical machinery of the sponge. Therefore, both methods were used for activity-guided purification of the CHCl_3_ subextract fractions (C6-C10). We also purified the inactive fraction C1 as the amino acid type signals observed in the ^1^H NMR spectrum were tracked to this fraction.

### 2.3. Purification and Structure Elucidation

**Figure 3:**
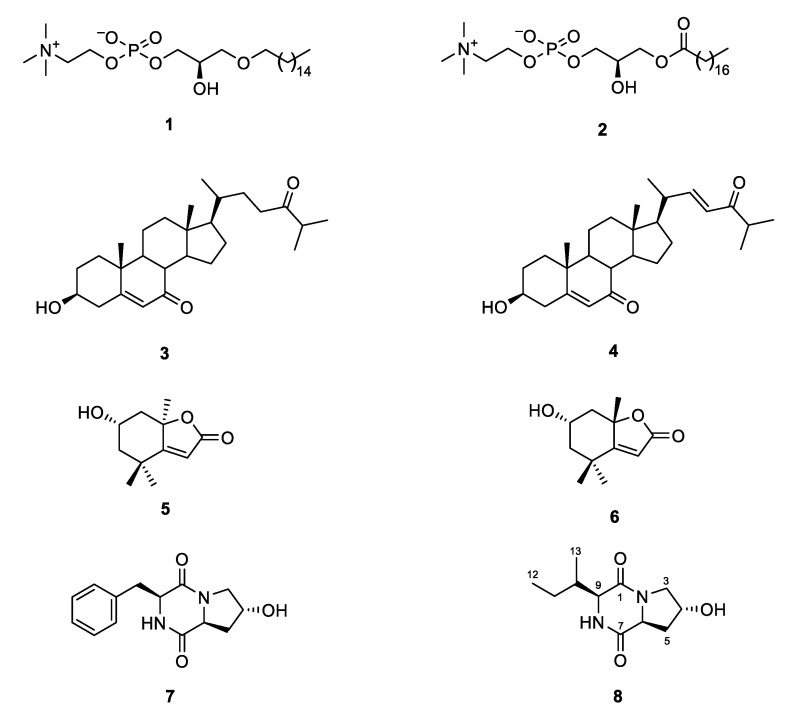
Chemical structure of compounds **1**–**8**.

The CHCl_3_ subextract with antibacterial activity was profiled by MN and ^1^H NMR spectroscopy. Reverse-phase (C18) flash column chromatography of the CHCl_3_ subextract yielded 12 fractions, out of which five showed antimicrobial activities (frs. C6–C10, Figure 1). All bioactive fractions were further submitted to RP-HPLC for purification. The chemical structures of the purified compounds were identified based on the comparison of their 1D and 2D NMR, HR-ESIMS data including MS^2^ fragmentation patterns and [α]_D_ data with those reported in the literature (Figures S4–S33). MN profiling was very useful for the targeted isolation of phospholipids 1-*O*-hexadecyl-*sn*-glycero-3-phosphocholine (*m/z* 482.339 [M + H]^+^) (**1**) [16] and 1-*O*-octadecanoyl-*sn*-glycero-3-phosphocholine (*m/z* 524.349 [M + H]^+^) (**2**) [17] (cluster A, Figure 2) as well as the steroids 3β-hydroxycholest-5-ene-7,24-dione (*m/z* 415.297 [M + H]^+^) (**3**) and (22*E*)-3β-hydroxycholesta-5,22-diene-7,24-dione (*m/z* 413.316 [M + H]^+^) (**4**) (cluster B, Figure 2) [20] from fractions C7–C10. In turn, ^1^H NMR spectroscopy was particularly helpful for guiding the isolation of the known (**7**) and the new (**8**) DKPs, which existed in the inactive fraction C1 and showed diagnostic resonances in the ^1^H NMR spectrum of the crude and the CHCl_3_ subextract (Figures S2 and S3) [22]. Finally, two monoterpenes loliolide (**5**) and 5-*epi*-loliolide (**6**) [23,24] which did not appear in the MN because of their low MS fragmentation intensity were also isolated from the active fraction C6 (Figure 3).

**Figure 4:**
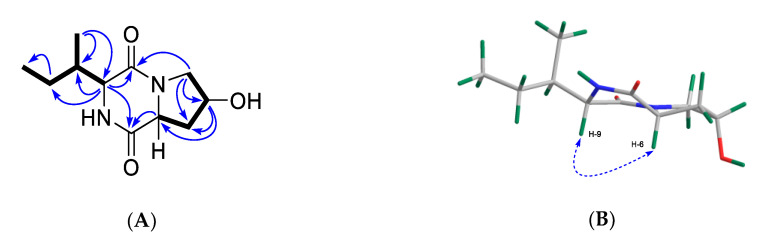
Key COSY (bold) and HMBC correlations H→C (arrows) (**A**) and key NOESY correlations (dashed line, **B**) observed for compound **8**.

Compound **8** was obtained as a colorless film. Its molecular formula C_11_H_18_N_2_O_3_ was deduced by HR-ESIMS (*m/z* 227.1407, [M + H]^+^) (Figure S33) indicating 4 degrees of unsaturation (DoU). The ^1^H and ^13^C NMR spectra (CD_3_OD) showed the presence of two carbonyl groups at *δ*_C_ 167.7 (C-1) and 172.7 (C-7), plus two α-methine protons at *δ*_H_ 4.48 (H-6) and 4.13 (H-9) that resonated at *δ*_C_ 58.3 and 61.2, respectively, suggesting **8** to be a dipeptide [22]. Additionally observed in the 1D NMR spectra (CD_3_OD) were one methine (*δ*_H_ 2.18, H-10; *δ*_C_ 36.9, C-10), one oxymethine (*δ*_H_ 4.47, H-4; *δ*_C_ 68.8, C-4) and three methylene protons [*δ*_H_ 3.72, 3.42, H_2_-3; *δ*_C_ 55.1, C-3; *δ*_H_ 2.29, 2.04, H_2_-5; *δ*_C_ 38.6, C-5; and *δ*_H_ 1.46, 1.33, H_2_-11; *δ*_C_ 25.4, C-11] as well as a secondary methyl (*δ*_H_ 1.08 d, *J* = 7.3 Hz, H_3_-13; *δ*_C_ 15.4, C-13) and a terminal methyl (*δ*_H_ 0.94 t, *J* = 7.4 Hz, H_3_-12; *δ*_C_ 12.5, C-12) group (Table 1). The ^1^H NMR spectrum acquired in DMSO-*d*_6_ revealed the presence of two exchangeable protons at *δ*_H_ 5.13 and 7.97 (Figure S25). Considering the molecular formula, DoU and comparison of the NMR and MS data with those of **7**, compound **8** was identified as a bicyclic diketopiperazine derivative [22]. The major difference between the two compounds was that **8** lacked the aromatic signals belonging to the phenylalanine residue found in **7**. To confirm the amino acid composition and identify the planar structure of **8** unambiguously, we acquired COSY and HMBC spectra of **8**. The COSY spectrum of **8** contained two spin systems (Figure 4A). The first one contained a proton network from C-3 to C-6 (Figure 4A). The strong COSY correlation of both H_2_-3 and H_2_-5 with the oxymethine proton H-4 indicated the position of the hydroxy-group at C-4. This was further confirmed by the diagnostic HMBC correlations of H_2_-3 with C-4 and C-5, of H-4 with C-6 and of H_2_-5 with C-3. The HMBC spectrum showed further correlations of both H_2_-5 and H-6 with the C-7 carbonyl group (Figure 4A). Hence, the first amino acid unit within the DKP scaffold was confirmed as hydroxyproline (Hyp). The second COSY spin system that included the proton network from H-9 to terminal H_3_-12 was identified as a secondary butyl group (Figure 4A). Therein, the α-methine proton (H-9) coupled with H-10 of the *sec-*butyl group spectrum, indicating the presence of an isoleucine (Ile) moiety as the second amino acid. This was further supported by the key HMBC correlations between H-9/C-11 and H_3_-13/C-9 (Figure 4A). Further HMBC correlations between H_2_-3/C-1 and H-9/C-7 completed the DKP ring system (Figure 4A). Thus, the planar structure of **8** was elucidated as cyclo(Hyp-Ile).

The relative configuration of the stereocenters within **8** was deduced with the aid of the NOESY spectrum, [α]_D_ value and comparison with data published in the literature. The observed NOE cross-peak between H-6 and H-9 indicated these protons to be on the same side (α) of the molecule (Figure 4B). The lack of any NOESY correlation between H-4 and H-6 suggested the opposite (β) orientation of H-4. The relative configuration of C-10 was assigned by comparison of the ^1^H and ^13^C NMR data of **8** with those of structurally similar compounds containing the isoleucine group [25,26]. A literature survey indicated that **8** shares identical NMR data with the known DKP, cyclo(*D*-*trans*-Hyp-*D*-Ile) isolated from the marine sponge *Stelletta* sp. [27], hence, these two compounds should share the same planar structure and relative configurations. However, compound **8** displayed a negative specific rotation value ([α]^20^_D_ -84, *c* 0.1, CHCl_3_) whereas the sign of the [α]_D_ value for the known metabolite cyclo(*D*-*trans*-Hyp-*D*-Ile) was positive ([α]^21^_D_ +12, *c* 0.025, CHCl_3_). Compound **8** was highly pure by NMR spectroscopy (Figures S25–S32) and HPLC-DAD-ELSD analysis on a chiral column, and the repeated [α]_D_ measurements gave the same negative optical sign and value. We conclude that compound **8** is the enantiomer of cyclo(*D*-*trans*-Hyp-*D*-Ile) [27] where both amino acid units are enantiomeric to the known compound. Hence, compound **8** is (-)-cyclo(*L*-*trans*-Hyp-*L*-Ile).

### 2.4. Bioactivity of Compounds **1**–**8**

All purified compounds (**1**–**8**) were tested against MRSA and Efm for their antimicrobial activities. Only 3β-hydroxycholest-5-ene-7,24-dione (**3**) showed moderate activity against MRSA with an IC_50_ value of 70 μg/mL. All other compounds were inactive at the highest test concentrations (100 μg/mL).

## 3. Discussion

The present study that involved an in-depth metabolomic study employing both HRMS/MS-based molecular networking and ^1^H NMR spectroscopy revealed the great chemical machinery of the marine sponge *A. sinoxea* originated from the Persian Gulf. Guided by two spectroscopic profiling methods in an integrated manner and combined with antimicrobial activity, eight compounds belonging to four different chemical families were isolated and chemically characterized. MN-based metabolomics also revealed the presence of several putatively new compounds, which we were not able to purify in sufficient quantities for spectroscopic analyses, further pointing out the untapped potential of *Axinella* sponges in marine biodiscovery.

Although the sponge genus *Axinella* is well-known for being a prolific producer of chemically diverse metabolites with promising bioactivities [5,6,7,8], *A. sinoxea* has remained a poorly studied species. Until now, only five reports dealing with chemical or biological investigations of *A. sinoxea* sponge are available in the literature [11,12,13,28,29]. All these studies were performed on sponge material collected from the Persian Gulf where the genus *Axinella* is represented by a single species, *A. sinoxea*. The very first study by Nazemi et al. in 2012 observed minimal antibacterial activity of the diethyl ether extract of *A. sinoxea* against *Bacillus subtilis spizizenii* and *S. aureus* [28]. Studies by Mahdian et al. (2015) [29] and Nazemi et al. (2020) [13] revealed moderate cytotoxicity of the nonpolar extracts of *A. sinoxea*, while Salimi et al. (2015) reported the selective apoptotic effect of standardized *A. sinoxea* methanolic extract in human chronic lymphocytic leukemia cells [11]. A very recent study by Jamebozorgi et al. (2019) investigated the fatty acid and steroid composition of *A. sinoxea* by GC-MS to identify several saturated and unsaturated fatty acids and eight known steroids belonging to cholesterol, ergosterol, stigmasterol and norgorgosterol subclasses [12]. None of these identified compounds were purified or tested for their bioactivities. However, the fatty acid and steroid containing fractions displayed a significant inhibitory activity against human cancerous and non-cancerous cell lines. In the present study, *A. sinoxea* extracts did not inhibit any of the six cancer cell lines or the non-cancerous human keratinocyte cell line HaCaT. To our knowledge, this is the first study employing in-depth metabolomics, bioactivity screening and an activity-guided isolation procedure on *A. sinoxea*. Additionally, for the first time, purified and chemically characterized natural products are being reported from the same sponge.

One major aim of this study was to profile the potential biological activities of *A. sinoxea* collected from the Persian Gulf in a number of in-house bioassays. Notably, the crude extract and its CHCl_3_-soluble portion displayed only in vitro antimicrobial activity against MRSA. The C18 flash chromatography fractions of the CHCl_3_-extract also inhibited the growth of Efm. However, after completion of the bioactivity-guided isolation process on the active fractions, none of the purified metabolites showed any effect against Efm, and only one compound (**3**) was moderately active towards MRSA. It is a common problem that the activity of natural extracts diminishes or becomes completely lost due to the lack of synergistic effects of various components after a long and tedious purification process of individual constituents. On the other hand, we have performed the very first and comprehensive metabolomics study on *A. sinoxea* with the aid of a modern and automated tool such as Molecular Networking (MN) in combination with a classical, but rapid and non-destructive chemical profiling method, ^1^H NMR spectroscopy. Molecular networking (MN) is a tandem mass spectrometry (MS^2^)-based metabolomics methodology performed on the open-access online platform Global Natural Products Social Molecular Networking (GNPS). It provides the ability to match tandem mass (MS^2^) spectrometry data from the users to GNPS spectral libraries to achieve an efficient automated dereplication. More importantly, MN is able to map out the chemical space of a biological sample by grouping similar metabolites that produce similar MS/MS fragmentation in one cluster [15]. Our research group has been using MN successfully to uncover the detailed chemical machinery of various marine macro- and micro-organisms followed by targeted isolation of new and bioactive marine natural products [14,30,31]. However, in the current study, MN provided limited information on the chemical composition of *A. sinoxea* and only two natural product families, i.e., phospholipids and steroids, which are the major components of sponge cell walls [32], have been annotated. This might be due to the low MS/MS fragmentation intensity or the lower abundances of the other metabolites in crude mixtures. The ^1^H NMR spectroscopy is, however, a universal method, where one can observe all types of proton resonances in an organic compound. The main limitation of the method is its low sensitivity, hence, ^1^H NMR spectra of natural extracts are generally dominated by the signals of primary molecules that are highly abundant in every organism. However, ^1^H NMR spectroscopy becomes more helpful after the solvent–solvent partition and fractionation of the crude extract, where most of the primary metabolites are removed and secondary metabolites that are generally produced in small quantities become readily visible for monitoring the purification steps.

It is well known that sponges contain many lipid compounds, such as sterols and phospholipids [33]. Since the first report of steroids from marine sponges by Bergman in 1949 [34], numerous reports have described conventional and unconventional steroids from demosponges with modifications on the tetracyclic nucleus or the side chain, including e.g., aminosteroids, sulfated polyhydroxysterols, side-chain-oxygenated sterols and sulfated steroid-amino acid conjugates [20,33,35,36,37]. *Axinella* sponges are known to have generally complex steroid profiles including steroids and nor-steroids [36,38,39]. The previous GC-MS study on *A. sinoxea* led to the identification of eight steroidal compounds: cholesta-5,22-dien-3β-ol, cholest-5-en-3β-ol, ergosta-5,22-dien-3β-ol, ergost-5-en-3β-ol, stigmasta-5,22-dien-3β-ol, γ-sitosterol, 33-norgorgosta-5,24(28)-dien-3β-ol and stigmasta-5,24(28)-dien-3β-ol [12]. The present study by UPLC-MS/MS annotated only cholestane type animal steroids, namely 7-ketocholesterol, 3β-hydroxycholest-5-ene-7,24-dione (**3**) and (22*E*)-3β-hydroxycholesta-5,22-diene-7,24-dione (**4**). The latter two compounds were also purified in sufficient quantities and characterized by spectroscopic methods. The sterols primarily have a functional role in sponges, as constituents of cell membranes and as metabolic precursors for the biosynthesis of diverse steroid (sub)classes [35,37]. Cholesterol is a membrane stabilizer, maintaining its integrity and adjusting its flexibility and permeability [40]. Sponge biomembranes also have a high content of phospholipids, including plasmalogens, alkylacyl and diacyl glycerophospholipids [40,41]. Djerassi et al. showed the pathways of phospholipid metabolism (from glycerol-3-phosphate precursor) in the demosponge *Microciona prolifera* back in 1991 [40]. The phospholipid composition of *Axinella verrucosa* has been identified to contain roughly a 1:1:1 mixture of phosphatidylethanolamines, phosphatidylglycerols and phosphatidylserine [42]. From *A. sinoxea*, we have dereplicated several 1-*O*-acyl-*sn*-glycerol or 1-*O*-alkyl-*sn*-glycerol type phosphatidylcholines, such as 1-*O*-heptadecyl-*sn*-glycero-3-phosphocholine, 1-(1*Z*-hexadecenyl)-*sn*-glycero-3-phosphocholine, 1-*O*-hexadecyl-*sn*-glycero-3-phosphocholine (**1**), 1-arachidoyl-2-hydroxy-*sn*-glycero-3-phosphocholine and 1-*O*-octadecanoyl-*sn*-glycero-3-phosphocholine (**2**). The compounds **1**–**2** were isolated and their structures were elucidated.

^1^H NMR profiling allowed us to observe the presence of DKPs (**7**, **8**), small cyclic peptide compounds, which did not appear on the MN of both crude and CHCl_3_ extracts of *A. sinoxea*. The first DKP (**7**) was previously purified from the sponge-associated yeast *Aureobasidium pullulans* [21], and from the endophytic bacterium *Nocardiopsis sp.* associated with the terrestrial plant *Mallotus nudiflorus* L. [22]. The known DKP, cyclo(*D*-*trans*-Hyp-*D*-Ile) that is the (+)-enantiomer of our compound **8** also originates from a marine sponge, *Stelletta sp.* [27]. New DKPs, such as verpacamides A−D type C_11_N_5_ DKPs relating to cyclo(Pro-Pro) and to cyclo(Pro-Prg), but also a number of known DKPs such as cyclo-(Pro-Thr), cyclo-(Pro-Ser), cyclo-(Pro-Ala), cyclo-(Pro-Leu), cyclo-(Pro-Val) and cyclo-(Pro-Gly) have been reported from the genus *Axinella* [8]. DKPs are the smallest cyclic peptides consisting of a minimum of two α-amino acids [43]. DKPs are an abundant class of biologically active natural compounds produced by a variety of marine microorganisms (bacteria, fungi, yeast) and macroorganisms such as sponges, tunicates and red algae [43,44]. Finally, we purified and identified two monoterpene lactone norisoprenoids, loliolide (**5**) and 5-*epi*-loliolide (**6**) from the CHCl_3_ subextract of *A. sinoxea*. As degraded lactonic carotenoids, they are very common in nature and have been reported from brown seaweeds, marine invertebrates, sediment as well as numerous terrestrial plants and animals, even from insects [45]. We have previously identified compounds **5** and **6** in the surface extract of the Baltic seaweed *Fucus vesiculosus* [46].

## 4. Materials and Methods

### 4.1. General Experimental Procedures

Optical rotations were measured using a Jasco P-2000 polarimeter (Jasco, Pfungstadt, Germany) equipped with a sodium lamp (589 nm). NMR spectra were obtained on a Bruker AV 600 spectrometer (600 and 150 MHz for ^1^H and ^13^C NMR, respectively) equipped with a triple resonance cryoprobe. The spectra were acquired at 298 K (25 °C). The samples were dissolved in 300 μL of deuterated solvent using a 5.0 mm Shigemi tube. The residual solvent signals were used as internal references: *δ*_H_ 7.26/*δ*_C_ 77.2 ppm (CDCl_3_), *δ*_H_ 3.31/*δ*_C_ 49.0 ppm (MeOD) and *δ*_H_ 2.50/*δ*_C_ 39.5 ppm (DMSO-*d*_6_). Tetramethylsilane (TMS) served as the internal standard. The ^1^H NMR spectrum (zg30) was acquired with 8 scans, while the ^13^C NMR spectrum (pulseprogram: zgpg30) was measured with 8192 scans. The COSY (pulseprogram: cosygpmfppqf) experiment was achieved with 2 scans and 1024 datapoints in the F1-dimension using non-uniform sampling, while NOESY (pulse program: noesygpphpp, 8 scans) was acquired with 512 in the F1 dimension. The HSQC spectrum (pulseprogram: hsqcedetgpsisp2.4) was acquired with 16 scans and 1024 datapoints in the F1-(C)-dimension using non uniform sampling, while the HMBC spectrum was run (pulseprogram: hmbcgplpndqf) with 24 scans and 400 datapoints in the F1-(C)-dimension. HR-MS/MS data were recorded on a Waters Xevo G2-XS QTof Mass Spectrometer (Waters^®^, Milford, MA, USA) coupled to a Waters Acquity I-Class UPLC system (Waters^®^, Milford, MA, USA). HR-ESIMS was recorded on a micrOTOF II-High-performance TOF-MS system (Bruker^®^, Billerica, MA, USA) equipped with an electrospray ionization source. Reversed phase C18 silica gel (50 μm, 65Å, Phenomenex, Torrance, CA, USA) was used for column chromatography (CC) to pre-fractionate the CHCl_3_ subextract. HPLC separations were performed on a VWR Hitachi Chromaster system coupled with a diode array detector, an autosampler and a pump combined in parallel with a VWR evaporative light scattering detector. Two solvents H_2_O + 0.1% FA (A) and acetonitrile + 0.1% FA (B) ULC/MS and HPLC grade were used as the mobile phase. Routine HPLC separations were performed on a semi-preparative C18 monolithic column (Onyx, 100 × 10 mm, Phenomenex) and an analytical synergi column (250 × 4.6 mm, Phenomenex). A chiral cellulose-1 column (Lux 5µ, 250 × 4.6 mm, Phenomenex) was used for checking the enantiopurity of each purified compound.

### 4.2. Sponge Material

The sponge sample was collected by scuba diving (−15 m) from a reef habitat around Larak Island (Persian Gulf) in June 2016. The sample was immediately frozen at −20 °C and transferred to the laboratory. It was identified through a scanning optical microscope, skeletal slides and dissociated spicule mounts based on the Hooper identification key [47]. A voucher specimen (De/Ax220) was deposited in the Persian Gulf and Oman Sea Ecological Center.

### 4.3. Extraction and Isolation

The sponge material (450 g, dry wt.) was desalted by extracting with Milli-Q Water (3 × 1.5 L) at room temperature. The sponge residue was then extracted with MeOH under agitation (3 × 1.5 L) to yield a yellowish crude organic extract (12.67 g). The methanolic extract was partitioned by a modified Kupchan method [14] to yield *n*-hexane (9.01 g), CHCl_3_ (2.63 g) and aq. MeOH (0.93 g) subextracts. The bioactive CHCl_3_-soluble fraction was subjected to C18 flash chromatography (25 µm, 250 × 20 mm i.d.) eluting with a step gradient of MeOH in H_2_O (0% to 100% MeOH) to afford 12 subfractions (C1–C12). The fraction C6 (58 mg) was subjected to a RP-HPLC separation on the Onyx Monolithic Semi-PREP C18 (column 5 µm, 100 × 10 mm i.d.) eluting with a gradient of H_2_O + 0.1% FA (A): MeCN + 0.1% FA (B) (75:25 to 70:30) at a flow rate of 2.5 mL/min to yield **5** (0.4 mg, *t*_R_ 5.5 min) and **6** (0.5 mg, *t*_R_ 7.5 min). The separation of subfraction C7 (94 mg) at the same conditions and at a gradient of 65% A to 60% A for 20 min afforded **3** (0.6 mg, *t*_R_ 8.2 min). The subfraction C8 (122 mg) was further purified by RP-HPLC using a gradient of 55% A to 50% A for 20 min to yield compound **4** (0.4 mg, *t*_R_ 10.4 min). The subfraction C10 (200 mg) was further separated by RP-HPLC analysis with a gradient of 25% A to 0% A for 30 min, to give **1** (1.1 mg, *t*_R_ 8.9 min) and **2** (1.2 mg, *t*_R_ 10.2 min). The subfraction C1 (15 mg) was also subjected to RP-HPLC on the Onyx Monolithic C18 (100 × 4.6 mm i.d.) column and eluted isocraticly with 95% A for 30 min at a flow rate of 1.5 mL/min to afford compounds **7** (1.9 mg, *t*_R_ 10.2 min) and **8** (1.2 mg, *t*_R_ 6.2 min). The enantiopurity of all compounds was checked by HPLC-DAD-ELSD on a chiral cellulose-1 analytical column using a gradient of MeCN (1% to 100% for 15 min at a flow rate of 1.5 mL/min).

*(-)-Cyclo(L-trans-Hyp-L-Ile)* (**8**): colorless oil. [α]^20^_D_ −84 (*c* 0.1, CHCl_3_); ^1^H NMR (CD_3_OD, 600 MHz) and ^13^C NMR (CD_3_OD, 150 MHz) see Table 1; HR-ESIMS *m/z* [M + H]^+^ 227.1407 (calcd. for C_11_H_19_N_2_O_3_ 227.1396).

### 4.4. UPLC-QToF-MS/MS Analysis

An ACQUITY UPLC I-Class System coupled to the Xevo G2-XS QToF Mass Spectrometer (Waters^®^, Milford, Massachusetts, USA) was used to analyze the crude MeOH extract, CHCl_3_ subextract and C18 subfractions, which was equipped with an electrospray ionization (ESI) source operating with a positive polarity at a mass range of *m/z* 50–1600 Da. The samples were dissolved in methanol at a concentration of 0.1 mg/mL and filtered through a 0.2 μm PTFE syringe filter (Carl Roth, Karlsruhe, Germany) and then 2 µL of each sample was injected into the system. The separation of compounds was achieved using a binary LC solvent system controlled by MassLynx^®^ (version 4.1) to analyze MS and MS^2^ data. An Acquity UPLC HSS T3 column (high-strength silica C18, 1.8 µm, 100 × 2.1 mm I.D., Waters^®^) was operated at 40 °C and eluted with H_2_O + 0.1% FA (A) and MeCN + 0.1% FA (B) ULC/MS grade at a flow rate of 0.6 mL/min with the following gradient: initial, 1%–100% B, 0–12 min; 100% B, 12–13 min and a column reconditioning phase until 15 min. The conditions of ESI were introduced as follows: capillary voltage: 0.8 kV, sample cone voltage: 40.0 V, source temperature: 150 °C, desolvation temperature: 550 °C, cone gas flow: 50 L/h, and desolvation gas flow: 1200 L/h. MS^2^ setting was kept at a collision energy (CE) of 30 eV.

### 4.5. Molecular Networking

The data of HR-ESI-LC-MS/MS of the crude extract were applied to create the network. At first, the raw MS/MS data were converted to mzXML format using the MSConvert software. The data were then uploaded to the Global Natural Products Social molecular networking (http://gnps.ucsd.edu) platform using FileZilla (https://filezilla-project.org/) to create the network on the online workflow at GNPS [15]. All MS^2^ peaks within +/− 17 Da of the precursor *m/z* were removed to filter for the data. MS^2^ spectra were filtered by selecting only the top 6 peaks in the +/− 50 Da window throughout the spectrum. All data were then networked with MS-Network with an MS^2^ fragment ion tolerance of 0.02 Da and a parent mass tolerance of 0.02 Da to create consensus spectra. Moreover, those including less than two spectra of the consensus spectra were deleted. A cosine score above 0.6 and more than three matched peaks were applied to create a network. Further, edges between two nodes were held in the network if and only if each of the nodes arrived in each other’s respective top 10 most similar nodes. The GNPS’ spectra library was used to filter the input data via library spectra due to similarities in the database. The input data must follow at least six match peaks with a score above 0.7 in library spectra to select as a matched output spectra. The output molecular networking data were visualized using the Cytoscape (ver. 3.61) [48].

### 4.6. Bioactivity Assays

The antimicrobial activity was initially tested in vitro at a final concentration of 100 µg/mL against the ESKAPE panel of multidrug-resistant bacterial human pathogens, including the Gram-positive bacteria *Enterococcus faecium* (Efm, DSM 20477) and methillicin-resistant *Staphylococcus aureus* (MRSA, DSM 18827), and the Gram-negative bacteria *Acinetobacter baumannii* (DSM 30007), *Klebsiella pneumoniae* (DSM 30104), *Escherichia coli* (DSM 1576) and *Pseudomonas aeruginosa* (DSM 1128). Moreover, the activity of the samples against two human pathogen yeasts, *Cryptococcus neoformans* (DSM 6973) and *Candida albicans* (DSM 1386), were performed. All test strains were bought from Leibniz Institute DSMZ-German Collection of Microorganisms and Cell Cultures, Braunschweig, Germany. The cultivation of bacteria took place in a TSB medium (0.5% NaCl, 1.2% tryptic soy broth), but *E. faecium* was grown in a M92 medium (3% trypticase soy broth, pH 7.0–7.2, 0.3% yeast extract). *C. albicans* was cultivated in M186/3 (0.1% malt extract, 0.17% peptone from soymeal, 0.3% glucose, 0.1% yeast extract) and *C. neoformans* was grown in M186 (1% glucose, 0.5% peptone from soymeal, 0.3% malt extract, 0.3 yeast extract). Overnight cultures of the test organisms were adjusted and diluted to an optical density (600 nm) of 0.01–0.03. To prepare the assay, a stock solution of 20 mg/mL in DMSO was prepared for the test samples and transferred into a 96-well microliter plate. A total of 200 µL of cell suspension cultures were added to each well. The incubation of inoculated microplates was performed for 5 h at 37 °C and 200 rpm (*E. faecium* without shaking), respectively, 28 °C and 200 rpm for 7 h for the *C. neoformans*. The detection of inhibitory effects was performed by adding 10 µL of a resazurin solution (0.3 mg/mL phosphate-buffered saline) to each cell and incubating again for 5–30 min. Then, 10 µL of a resazurin solution was added to the microplates and incubated again for 5–30 min before the fluorescence signal (560 nm/590 nm) was read by the microplate reader (Tecan Infinite M200). For *E. faecium* the pH indicator bromocresol purple was used to determine the acidification caused by growing. For *R. solanacearum* and *C. neoformans*, the optical density at 600 nm after the incubation time was recorded using the microplate reader. Chloramphenicol was the positive control used for the bacteria, nystatin for *C. albicans* and amphotericin B for *C. neoformans*. The IC_50_ values were calculated by Excel to determine the concentration that shows 50% inhibition of the viability.

The anticancer activity was assessed against six human cancer cell lines, lung carcinoma cell line A549 (CLS, Eppelheim, Germany), colorectal adenocarcinoma cell line HT29 (DSMZ, Braunschweig, Germany), malignant melanoma cell line A375 (CLS, Eppelheim, Germany), liver cancer cell line Hep G2 (DSMZ, Braunschweig, Germany), colon cancer cell line HCT116 (DSMZ, Braunschweig, Germany), human breast cancer line MDA-MB231 (CLS, Eppelheim, Germany) and the non-cancerous human keratinocyte cell line HaCaT (CLS, Eppelheim, Germany). The cytotoxic activity was evaluated by monitoring the metabolic activity at 37 °C under a humidified atmosphere and 5% CO_2_ in CellTiterBlue Cell Viability Assay (Promega, Mannheim, Germany). The cells of HT29, Hep G2 and HCT116 were cultivated in an RPMI 1640 medium (Life Technologies, Darmstadt, Germany) with 10% fetal bovine serum, 100 U/mL penicillin and 100 mg/mL streptomycin supplemented at 37 °C and 5% CO_2_, A549 cell in DMEM: Ham’s F12 medium (1:1) supplemented with 15 mM HEPES as well as A375 and HCT116 cells in a DMEM medium supplemented with 4.5 g/L D-Glucose and 110 mg/L sodium pyruvate. The seeding of cells was carried out in 96-well plates at a concentration of 10,000 cells per well for bioactivity tests. For the experimental procedure, a stock solution of 40 mg/mL in DMSO was prepared for each extract. Furthermore, 100 µL of fresh medium containing the test samples was replaced with the medium in the cells and then all of the samples were prepared in duplicates. Growth media and 0.5% DMSO were used as negative controls, while doxorubicin served as a positive control. The samples were added to the cells, which were incubated for 24 h at 37 °C. The assay was performed according to the manufacturer’s instructions (Promega), and measured using the microplate reader Tecan Infinite M200 (Tecan, Crailshaim, Germany) at an excitation of 560 nm and an emission of 590 nm. The IC_50_ values were determined by 50% inhibition of cell viability according to negative control (no compound) by Excel. Doxorubicin was the positive control.

## 5. Conclusions

By employing a state-of-the-art automated dereplication strategy such as MN in combination with a traditional chemical profiling method, ^1^H NMR spectroscopy, we were able to unravel the chemical machinery of the Persian Gulf sponge *A. sinoxea*. This approach, together with antimicrobial activity assessments, enabled the purification and characterization of eight compounds from four different natural product classes, which have never been reported from *A. sinoxea* before. We have also identified a new DKP (**8**), which is the enantiomer of another sponge-derived DKP. The known cholesterol derivative **3** exerted moderate anti-MRSA activity, and this is the first report of such activity observed with this compound. Overall, an integrated approach employing several metabolomics/profiling techniques in a complementary manner can assist the identification and investigation of the real chemical diversity of marine organisms to guide the discovery of their new and bioactive constituents. This study further confirms the untapped potential of marine sponges in the Persian Gulf for marine biodiscovery and stimulates future studies on these organisms.

## Figures and Tables

**Table 1:** ^1^H NMR (600 MHz) and ^13^C NMR (150 MHz) data of compound **8** in MeOD.

Position	*δ*_H_, Mult. (*J* in Hz)	*δ*_C_, Type
1	-	167.7 (C)
3	3.72 (dd 12.9, 4.6) 3.42 (m)	55.1 (CH_2_)
4	4.47 (m)	68.8 (CH)
5	2.29 (m) 2.04 (ddd 13.3, 11.7, 4.3)	38.6 (CH_2_)
6	4.48 (m)	58.3 (CH)
7	-	172.7 (C)
9	4.13 (br s)	61.2 (CH)
10	2.18 (m)	36.9 (CH)
11	1.46 (m) 1.33 (m)	25.4 (CH_2_)
12	0.94 (t 7.4)	12.5 (CH_3_)
13	1.08 (d 7.3)	15.4 (CH_3_)

## Supplementary Materials

The following are available online at https://www.mdpi.com/1660-3397/18/7/366/s1. Putative identification of known compounds in the Global MN of the crude extract. Molecular networking analysis of the CHCl_3_ subextract. NMR and HR-ESIMS spectra of compounds **1**–**8**.
