# Supplementary material for: Integrating Molecular Networking and 1H NMR Spectroscopy for Isolation of Bioactive Metabolites from the Persian Gulf Sponge Axinella sinoxea
Source: Mar Drugs. 2020 Jul 16;18(7):366. doi: 10.3390/md18070366 (PMC7404180; doi:10.3390/md18070366)
Supplement: Supplementary file 1 [file marinedrugs-18-00366-s001.pdf]

Supporting Information

Integrating Molecular Networking and ^{1}H NMR Spectroscopy for Isolation of Bioactive Metabolites from the Persian Gulf Sponge *Axinella sinoxea*

Reza Mohsenian Kouchaksaraee [1,2], Mahdi Moridi Farimani [1,*], Fengjie Li [2], Melika Nazemi [3] and Deniz Tasdemir [2,4,*]

[1] Department of Phytochemistry, Medicinal Plants and Drugs Research Institute, Shahid Beheshti University, G. C., Evin, 1983969411 Tehran, Iran; r_mohsenian@sbu.ac.ir

[2] GEOMAR Centre for Marine Biotechnology (GEOMAR-Biotech), Research Unit Marine Natural Products Chemistry, GEOMAR Helmholtz Centre for Ocean Research Kiel, Am Kiel-Kanal 44, Kiel 24106, Germany; fli@geomar.de

[3] Persian Gulf and Oman Sea Ecological Center, Iranian Fisheries Science Research Institute, Agricultural Research, Education and Extension Organization (AREEO), 7916793165 Bandar Abbas, Iran; melikanazemi@yahoo.com

[4] Faculty of Mathematics and Natural Sciences, Kiel University, Christian-Albrechts-Platz 4, Kiel 24118, Germany

* Correspondence: dtasdemir@geomar.de; Tel.: +49-431-6004430
m_moridi@sbu.ac.ir; Tel.: +98-21-29904043

Content	Page No.

**Table S1.** Putative identification of known compounds in the global MN of the crude MeOH extract and $CHCl_3$ subextract of *Axinella sinoxea*	4
**Figure S1.** Global molecular networking of the $CHCl_3$ subextract (KC) of *Axinella sinoxea*	6
**Figure S2.** ^{1}H NMR spectrum of the crude extract of *Axinella sinoxea* (600 MHz, CD_3OD)	7
**Figure S3.** ^{1}H NMR spectrum of the $CHCl_3$ subextract (KC) of *Axinella sinoxea* (600 MHz, CD_3OD)	7
**Figure S4.** ^{1}H NMR spectrum of compound **1** (600 MHz, CD_3OD)	8
**Figure S5.** ^{13}C NMR spectrum of compound **1** (150 MHz, CD_3OD)	8
**Figure S6.** HR-ESIMS spectrum of compound **1**	9
**Figure S7.** ^{1}H NMR spectrum of compound **2** (600 MHz, CD_3OD)	10
**Figure S8.** ^{13}C NMR spectrum of compound **2** (150 MHz, CD_3OD)	10
**Figure S9.** HR-ESIMS spectrum of compound **2**	11
**Figure S10.** ^{1}H NMR spectrum of compound **3** (600 MHz, $CHCl_3$)	12
**Figure S11.** ^{13}C NMR spectrum of compound **3** (150 MHz, $CHCl_3$)	12
**Figure S12.** HR-ESIMS spectrum of compound **3**	13
**Figure S13.** ^{1}H NMR spectrum of compound **4** (600 MHz, $CHCl_3$)	14
**Figure S14.** HMBC spectrum of compound **4** (600 MHz, $CHCl_3$)	14
**Figure S15.** HR-ESIMS spectrum of compound **4**	15
**Figure S16.** ^{1}H NMR spectrum of compound **5** (600 MHz, CD_3OD)	16
**Figure S17.** ^{13}C NMR spectrum of compound **5** (150 MHz, CD_3OD)	16
**Figure S18.** HR-ESIMS spectrum of compound **5**	17
**Figure S19.** ^{1}H NMR spectrum of compound **6** (600 MHz, CD_3OD)	18
**Figure S20.** ^{13}C NMR spectrum of compound **6** (150 MHz, CD_3OD)	18
**Figure S21.** HR-ESIMS spectrum of compound **6**	19
**Figure S22.** ^{1}H NMR spectrum of compound **7** (600 MHz, CD_3OD)	20
**Figure S23.** ^{13}C NMR spectrum of compound **7** (150 MHz, CD_3OD)	20
**Figure S24.** HR-ESIMS spectrum of compound **7**	21
**Figure S25.** ^{1}H NMR spectrum of compound **8** (600 MHz, DMSO-d_6)	22
**Figure S26.** ^{13}C NMR spectrum of compound **8** (150 MHz, DMSO-d_6)	22
**Figure S27.** ^{1}H NMR spectrum of compound **8** (600 MHz, CD_3OD)	23
**Figure S28.** ^{13}C NMR spectrum of compound **8** (150 MHz, CD_3OD)	23

Figure S29. HSQC spectrum of compound **8** (150/600 MHz, CD_3OD) 24

Figure S30. HMBC spectrum of compound **8** (150/600 MHz, CD_3OD) 24

Figure S31. COSY spectrum of compound **8** (600 MHz, CD_3OD) 25

Figure S32. NOESY spectrum of compound **8** (600 MHz, CD_3OD) 25

Figure S33. HR-ESIMS spectrum of compound **8** 26

References 27

Table S1. Putative identification of known compounds in the global MN of the crude MeOH extract and $CHCl_3$ subextract of *Axinella sinoxea.*

Cluster A and A1

No.	t_R (min)	Parent mass *m/z*	Putative ID	Molecular formula of the *m/z* [M + H]+	Δ ppm	Key MS² fragments	LoA*	Reference
1[b]	8.3	468.321	1-*O*-pentadecyl-*sn*-glycero-3-phosphocholine	$C_{23}H_{51}NO_6P$	2.1	450.4125; 285.0731; 184.0750; 104.1051	3	1
3[a]	8.2	480.321	1-(1*Z*-hexadecenyl)-*sn*-glycero-3-phosphocholine	$C_{24}H_{51}NO_6P$	1.1	462.3144; 297.2121; 184.3345; 104.3651	3	2
4[a]	9.0	482.339	1-*O*-hexadecyl-*sn*-glycero-3-phosphocholine	$C_{24}H_{53}NO_6P$	1.2	464.2720; 299.2960; 184.1212; 104.2348	5	3
5[b]	8.7	494.309	1-(1*Z*-heptadecenyl)-glycero-3-phosphocholine	$C_{25}H_{53}NO_6P$	-1.5	476.5814; 311.5471; 184.4714; 104.7451	2	4
6[a]	8.7	496.314	1-*O*-heptadecyl-*sn*-glycero-3-phosphocholine	$C_{25}H_{55}NO_6P$	0.8	478.3310; 313.2881; 184.3414; 104.3257	3	5
7[b]	11.4	508.352	1-(1*Z*-octadecenyl)-*sn*-glycero-3-phosphocholine	$C_{26}H_{55}NO_6P$	-1.1	490.8764; 325.4761; 184.5487; 104.5541	2	2
8[b]	10.1	510.369	1-*O*-octadecyl-*sn*-glycero-3-phosphocholine	$C_{26}H_{57}NO_6P$	2.1	492.4541; 327.5151; 184.3561; 104.3821	3	6
9[b]	8.4	512.374	1-*O*-(2-methoxyhexadecyl)-*sn*-glycero-3-phosphocholine	$C_{25}H_{55}NO_7P$	3.2	494.3871; 329.4542; 184.3320; 104.3224	2	7
10[a]	9.9	524.349	1-*O*-octadecanoyl-*sn*-glycero-3-phosphocholine	$C_{26}H_{55}NO_7P$	0.9	506.3620; 341.3040; 184.0740; 104.1070	5	8
11[b]	10.5	538.347	1-*O*-nonadecanoyl-*sn*-glycero-3-phosphocholine	$C_{27}H_{57}NO_7P$	1.3	520.3945; 510.3910; 355.4573; 184.0531; 104.1273	3	8
12[a]	9.0	552.379	1-arachidoyl-2-hydroxy-*sn*-glycero-3-phosphocholine	$C_{28}H_{59}NO_7P$	2.2	534.3920; 369.3310; 184.1312; 104.1136	3	9

Cluster B and B1

No.	t_R (min)	Parent mass *m/z*	Putative ID	Molecular formula pf the *m/z* [M + H]+	Δ ppm	Key MS² fragments	LoA*	Reference

1[b]	7.4	385.428	(22*E*)-3β-hydroxy-26,27-bisnorcholesta-5,22-diene-7,24-dione	$C_{25}H_{37}O_3$	1.7	367.2640; 349.2560; 315.2370; 287.2001; 251.1752; 157.1041; 99.0800	2	10
2[b]	7.8	387.264	3β-hydroxy-26,27-bisnorcholesta-5-ene-7,24-dione	$C_{25}H_{39}O_3$	1.2	369.2454; 351.2670; 269.2850; 255.2280; 157.1012; 175.1290, 99.1812	2	10
3[b]	10.1	399.301	(22*E*)-3β-hydroxycholesta-5,22-dien-24-one	$C_{27}H_{43}O_2$	-0.6	381.3201; 363.3225; 283.2678; 269.2138; 173.1012; 111.1167; 69.0690	2	10
4[a]	12.1	401.316	7-ketocholesterol	$C_{27}H_{45}O_2$	0.6	383.3300; 365.3230; 271.2050; 253.1241; 175.1108; 159.1214; 81.05415	3	11
5[b]	11.6	413.316	(22*E*)-3β-hydroxycholesta-5,22-diene-7,24-dione	$C_{27}H_{41}O_3$	-0.6	395.4581; 377.3200; 315.2341; 291.8412; 175.1115; 121.3245; 83.0530	5	10
6[b]	9.1	415.297	3β-hydroxycholesta-5-ene-7,24-dione	$C_{27}H_{43}O_3$	0.3	397.3110; 379.3145; 361.2912; 293.2341; 203.5412; 127.1218; 109.5471;	5	10

*: LoA (Level of Assignment); 1: Accurate mass matched to database-Tentative assignment, 2: Accurate mass matched to database and tandem MS spectrum matched to *in silico* fragmentation pattern, 3: Tandem MS spectrum matched to database or literature, 4: RT matched to standard compound, 5: MS/MS spectrum matched to standard compound.

[a]: Annotation via automated dereplication; [b]: Annotation via manual dereplication

Automated dereplication was performed on GNPS platform. Manual dereplication was performed considering the parent mass, biological source, retention time, elemental composition analysis, and predicated fragmentation patterns.

The MS^2 fragmentation pattern of a molecule was predicated on the Competitive Fragmentation Modeling for Metabolite Identification (CFM-ID) platform (http://cfmid.wishartlab.com) and compared with our experimental data.

Figure S1. Global molecular networking of the CHCl3 subextract (KC) of *Axinella sinoxea*.

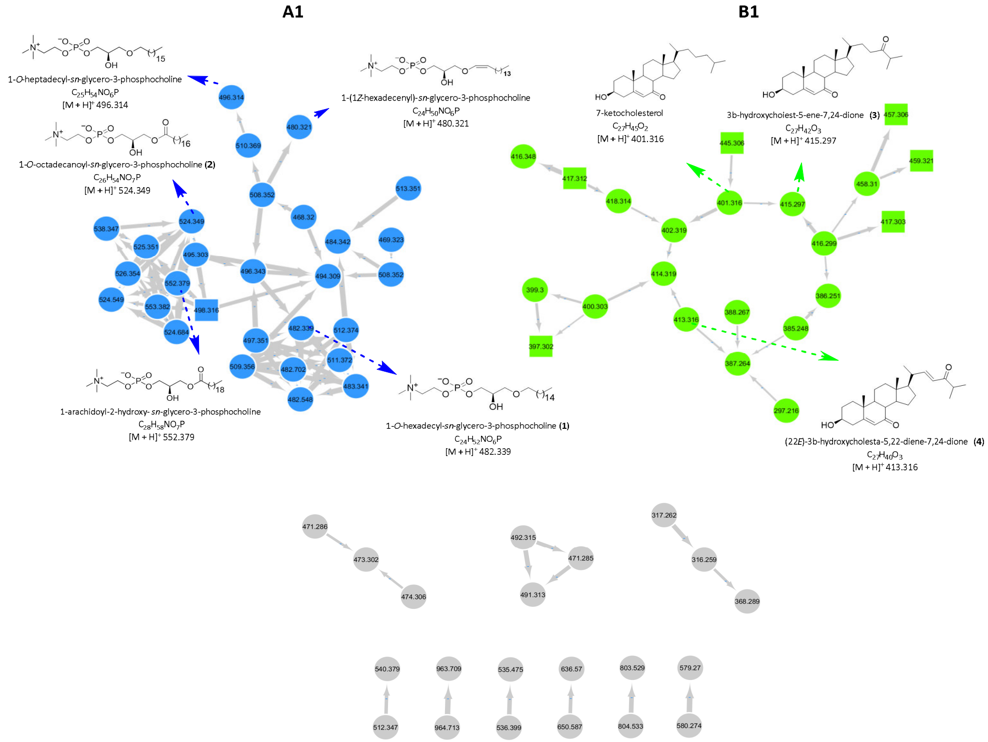

Blue nodes represent phospholipids and green nodes represent steroids. The thickness of the edges indicates the similarity of the nodes. The numbers within the nodes represent parent ions. Square nodes represent putatively new compounds. Gray nodes could not be annotated to any known chemical classes either by automated or manual dereplication. All molecules from clusters **A1** and **B1** were annotated to be the same as those in the global MN of the crude extract of *Axinella sinoxea* (Table S1).

Figure S2. ^{1}H NMR spectrum of the crude extract of *Axinella sinoxea* (600 MHz, CD_3OD)

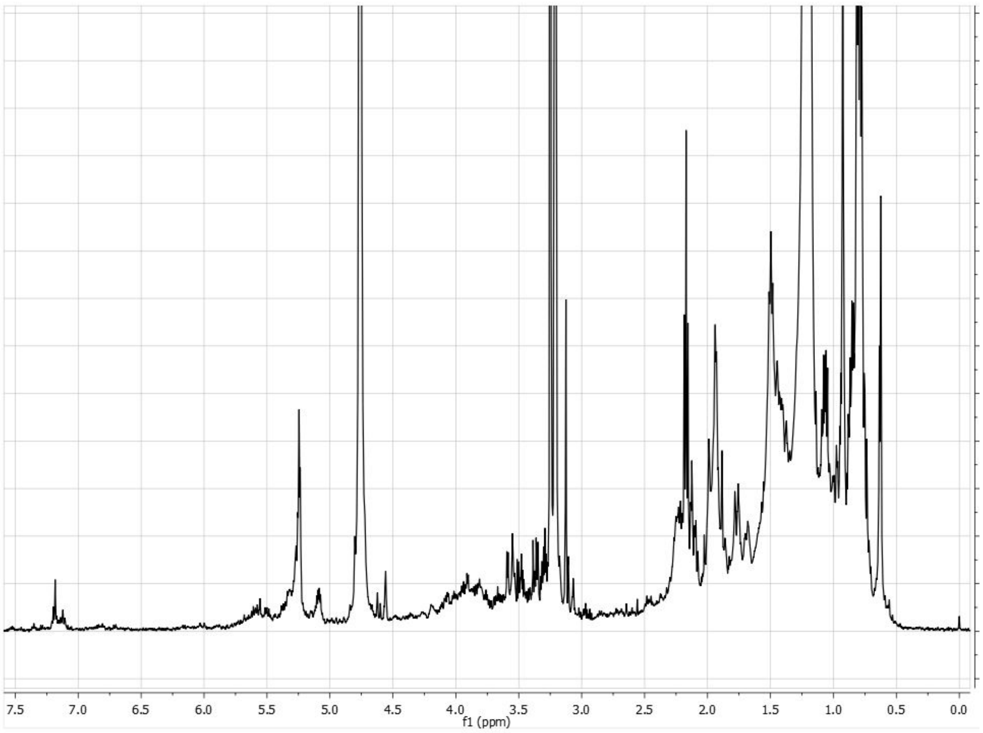

Figure S3. ^{1}H NMR spectrum of the $CHCl_3$ subextract (KC) of *Axinella sinoxea.* (600 MHz, CD_3OD)

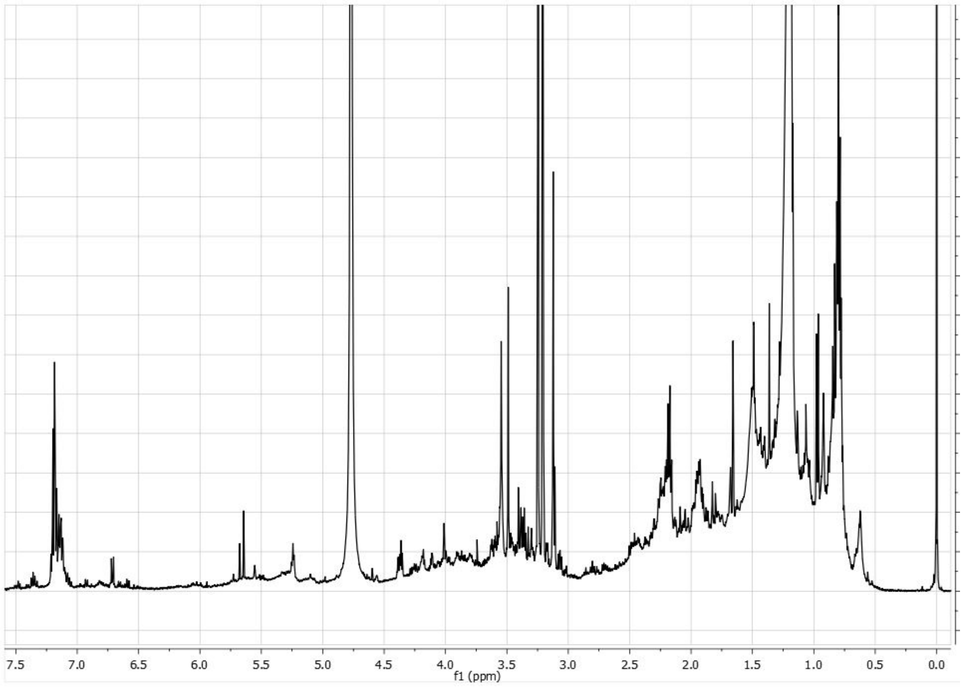

Figure S4. ^{1}H NMR spectrum of compound **1** (600 MHz, CD_3OD).

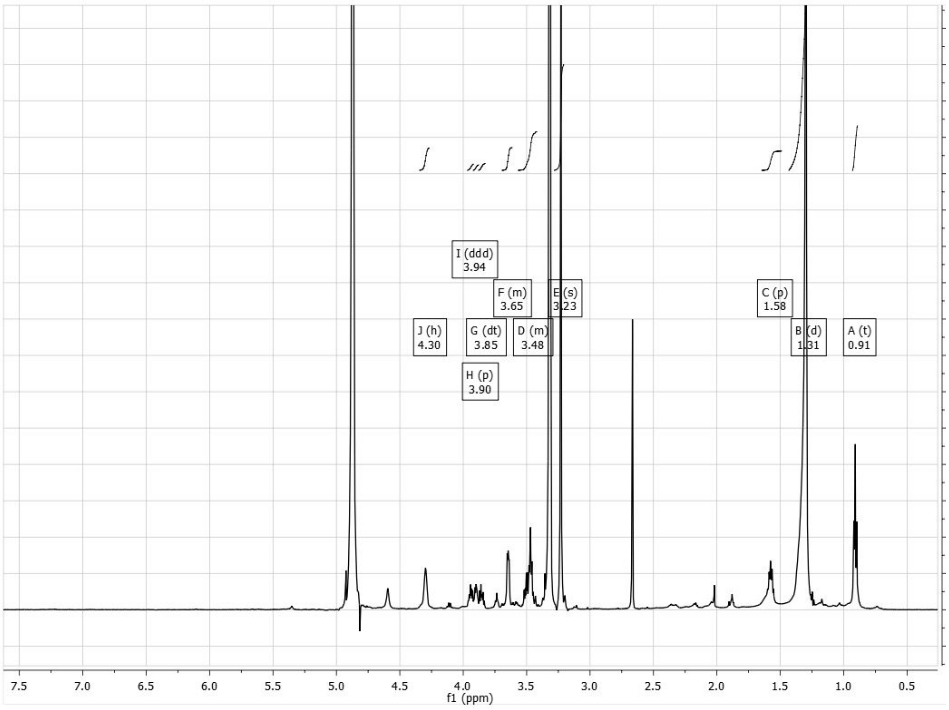

Figure S5. ^{13}C NMR spectrum of compound **1** (150 MHz, CD_3OD).

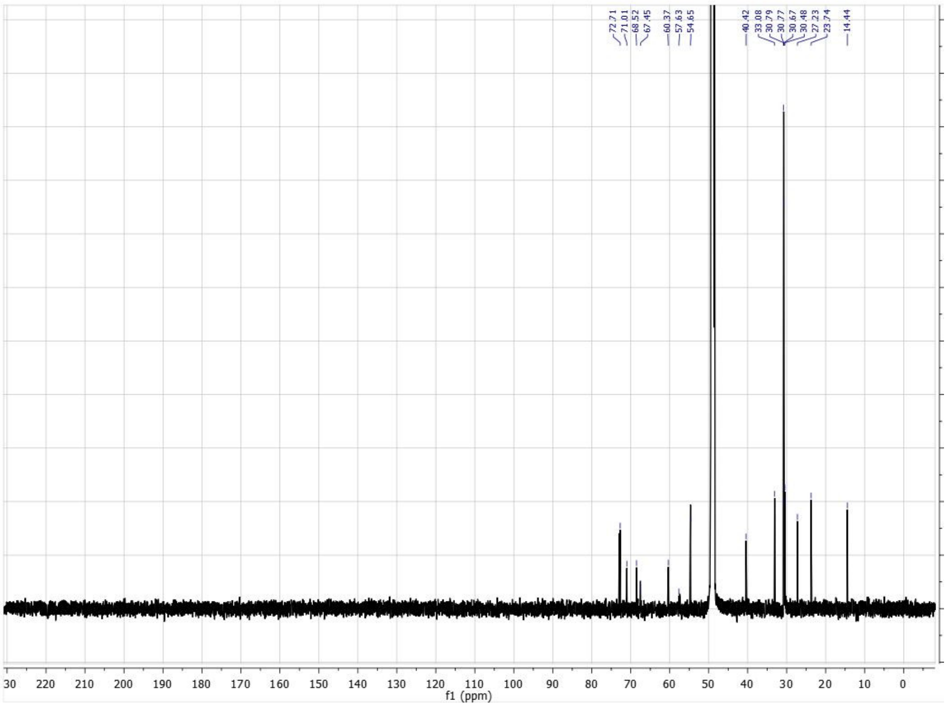

Figure S6. HR-ESIMS spectrum of compound **1**.

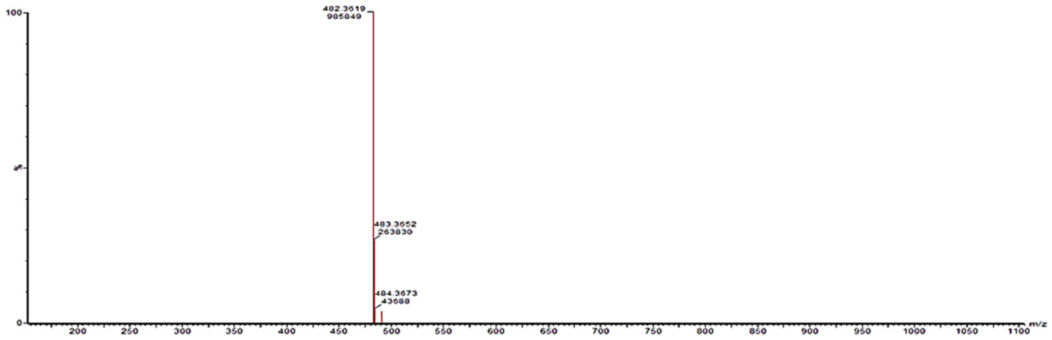

Figure S7. ^{1}H NMR spectrum of compound **2** (600 MHz, CD_3OD).

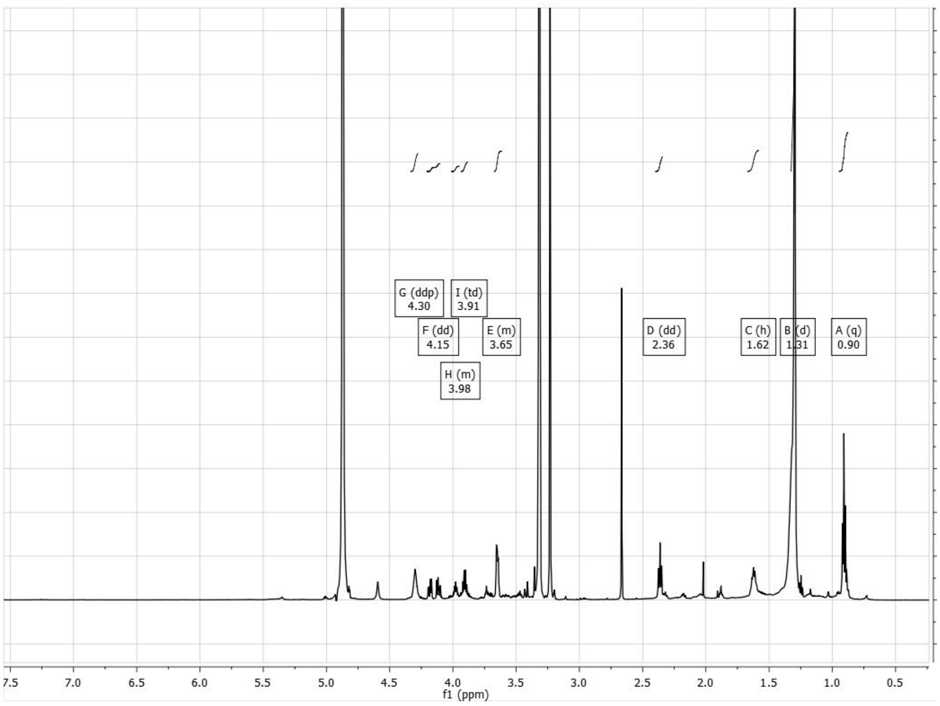

Figure S8. ^{13}C NMR* spectrum of compound **2** (150 MHz, CD_3OD).

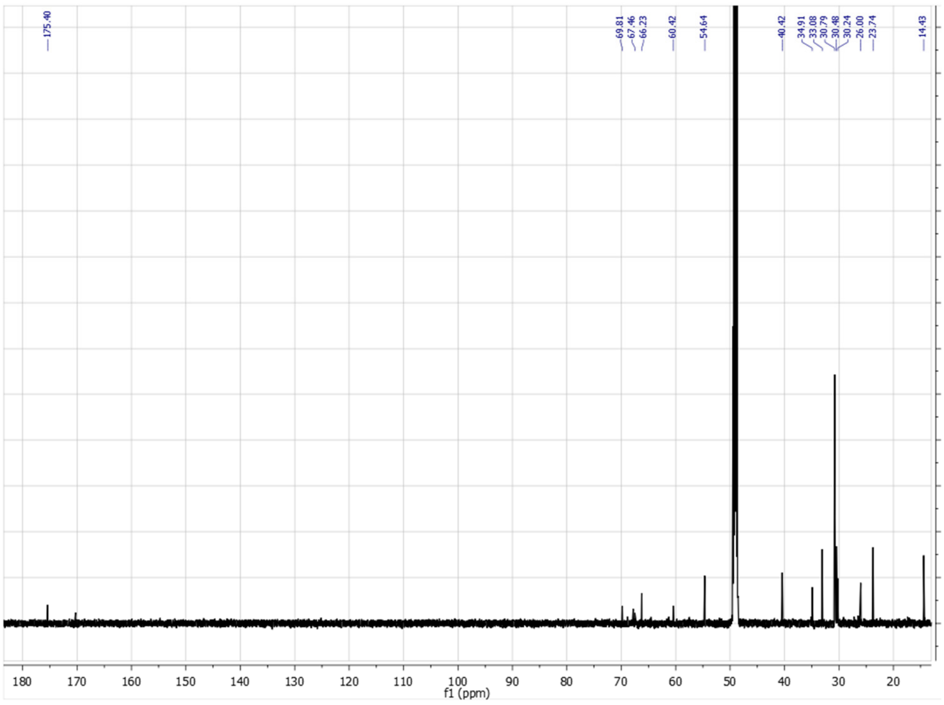

Figure S9. HR-ESIMS spectrum of compound **2**.

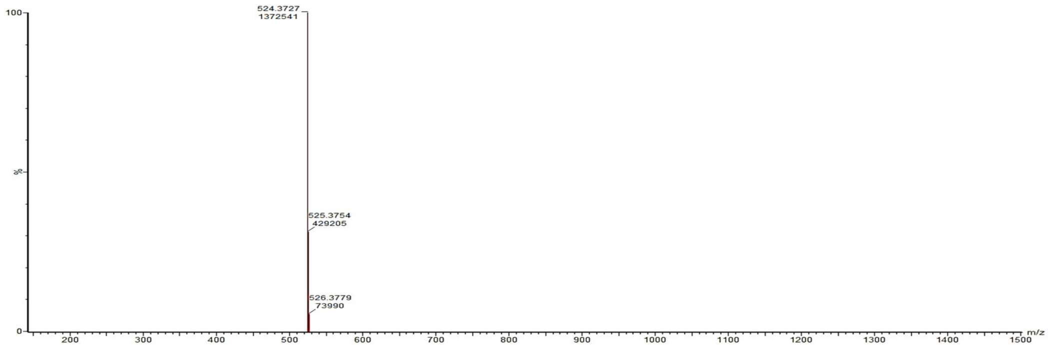

Figure S10. ^{1}H NMR spectrum of compound **3** (600 MHz, $CHCl_3$).

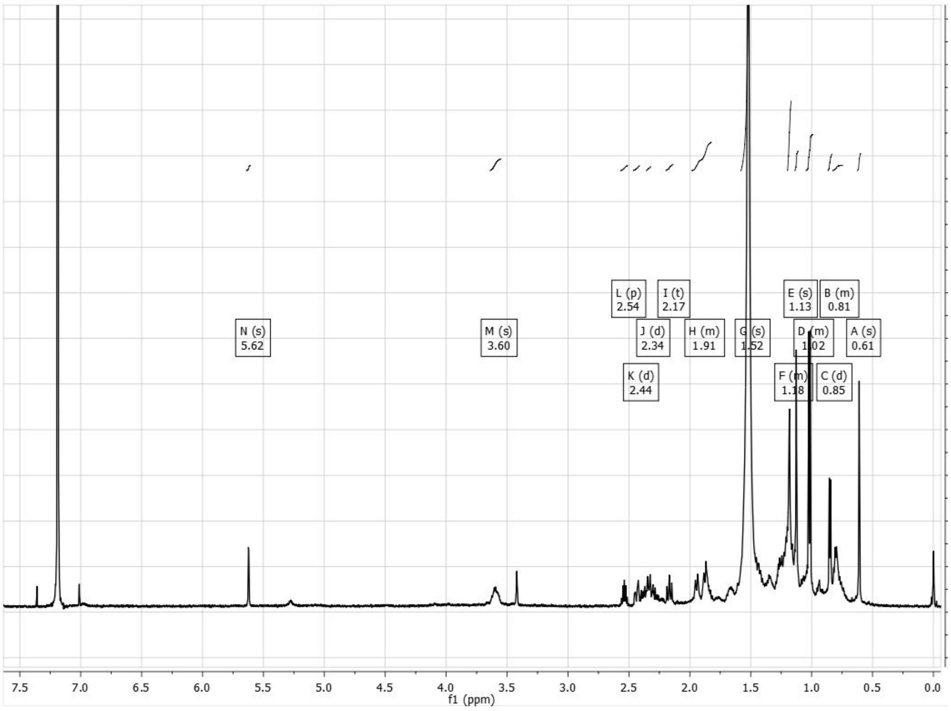

Figure S11. ^{13}C NMR spectrum of compound **3** (150 MHz, $CHCl_3$).

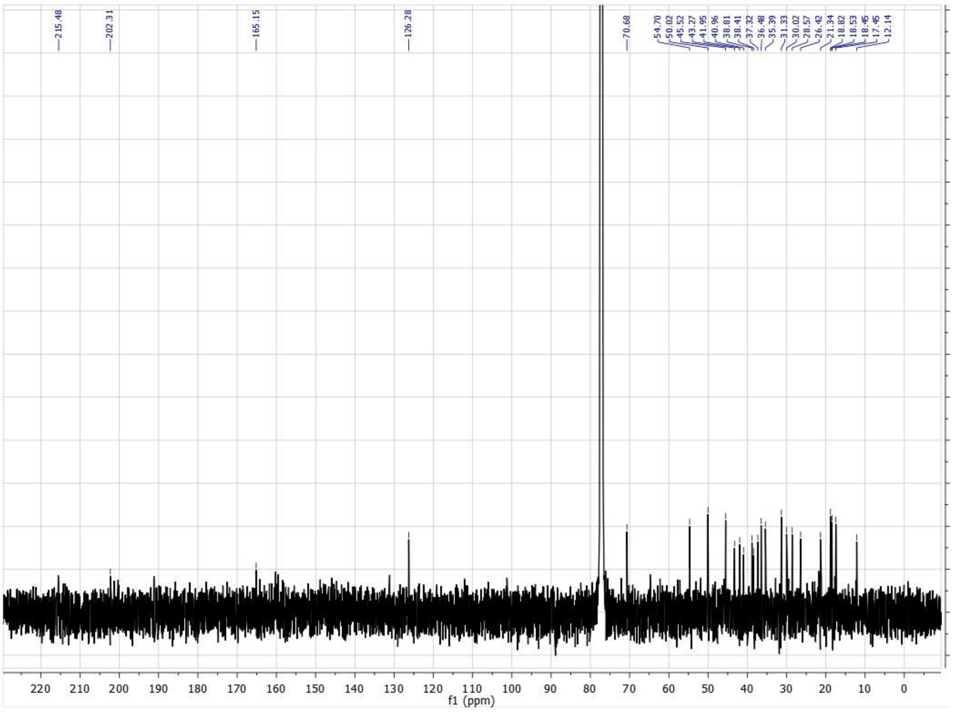

Figure S12. HR-ESIMS spectrum of compound **3**.

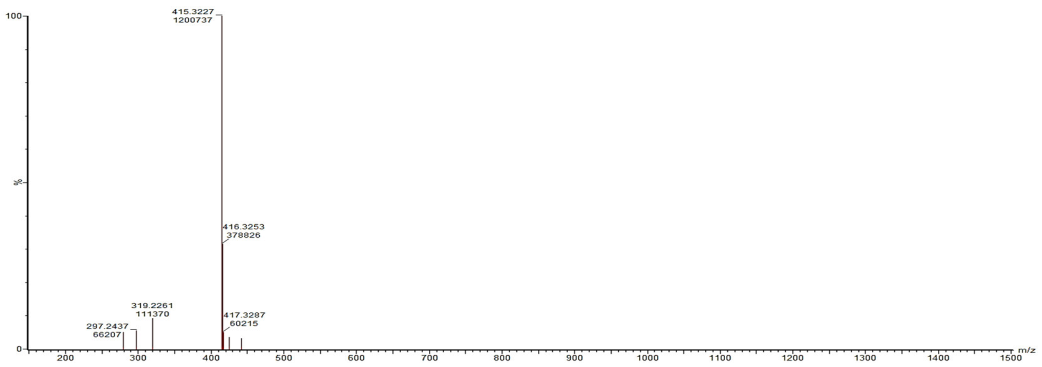

Figure S13. ^{1}H NMR spectrum of compound **4** (600 MHz, $CHCl_3$).

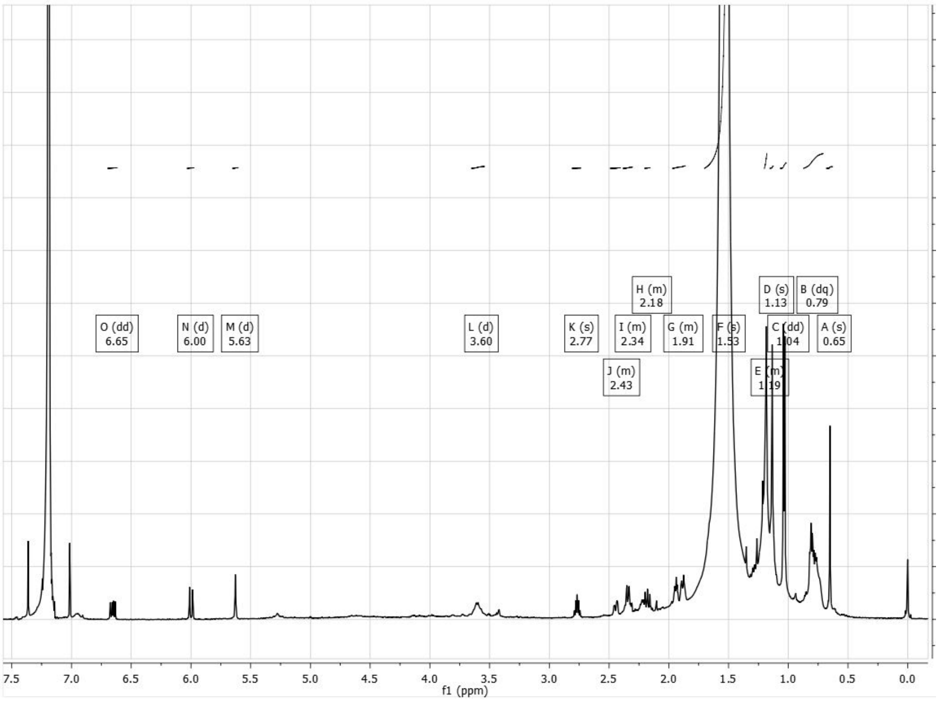

Figure S14. HMBC spectrum of compound **4** (600 MHz, $CHCl_3$).

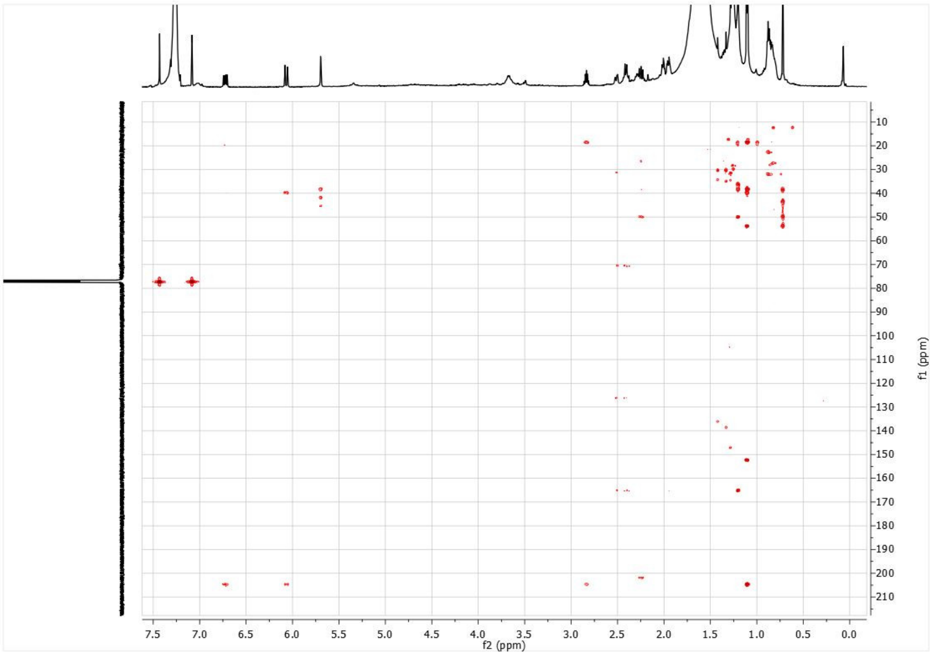

Figure S15. HR-ESIMS spectrum of compound **4**.

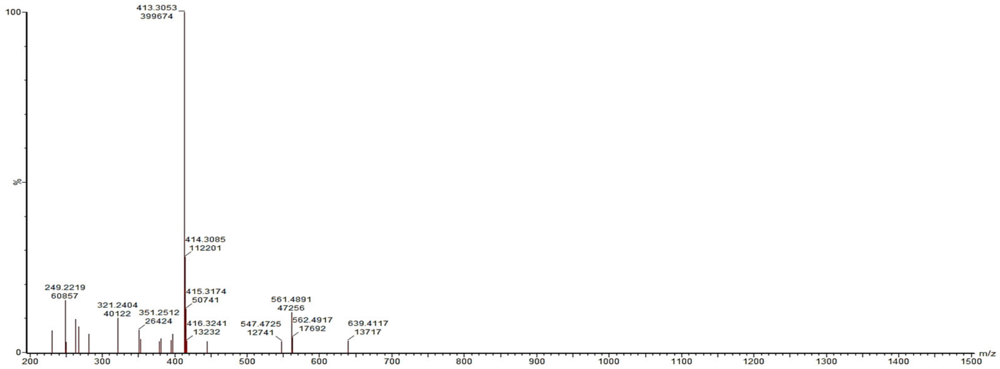

Figure S16. ^{1}H NMR spectrum of compound **5** (600 MHz, CD_3OD).

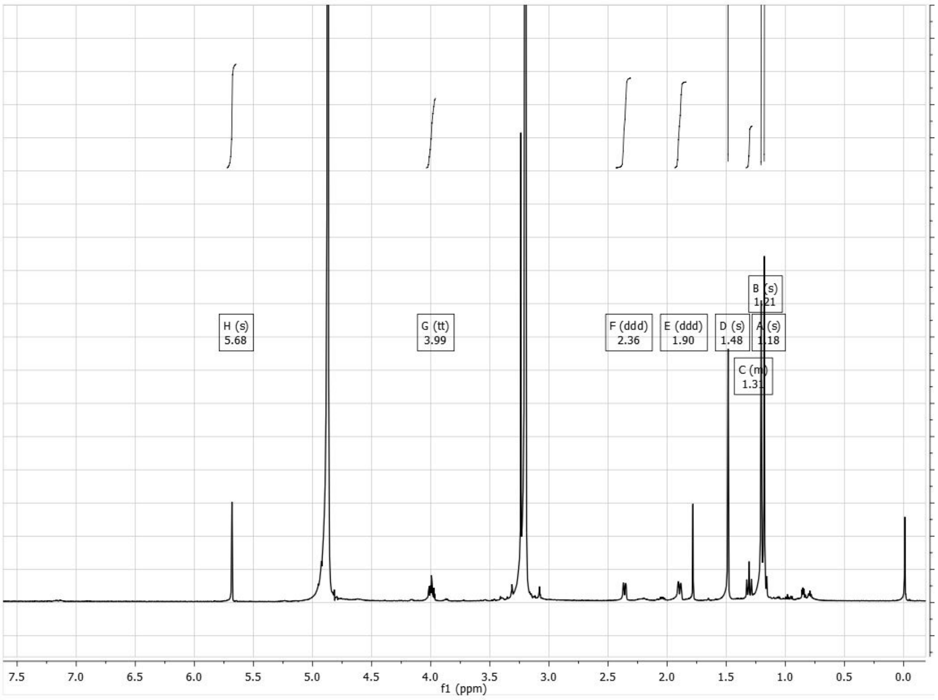

Figure S17. ^{13}C NMR spectrum of compound **5** (150 MHz, CD_3OD).

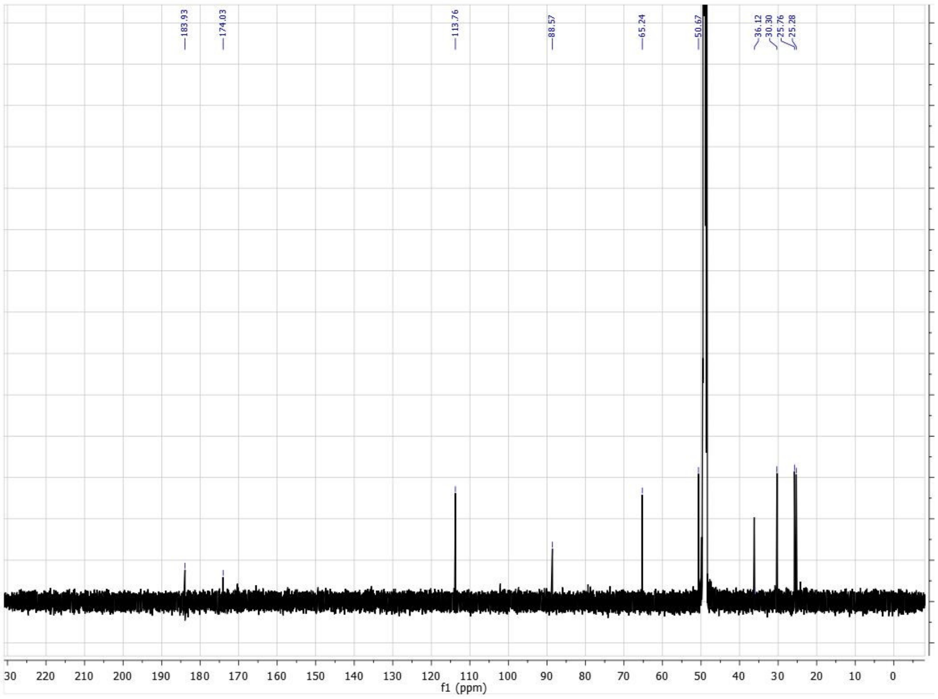

Figure S18. HR-ESIMS spectrum of compound **5**.

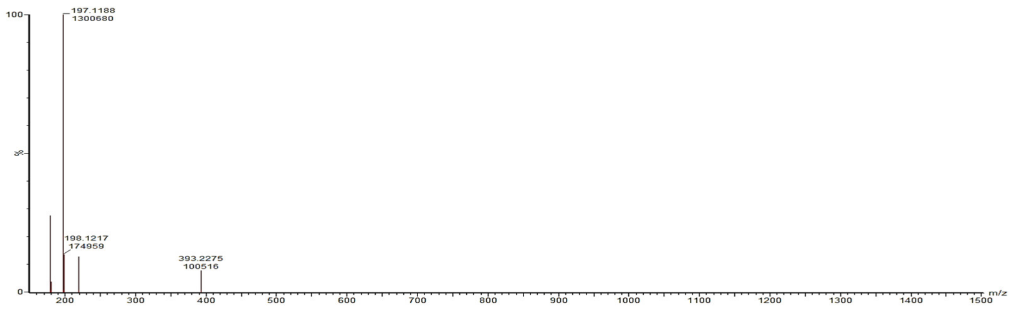

Figure S19. ^{1}H NMR spectrum of compound **6** (600 MHz, CD_3OD).

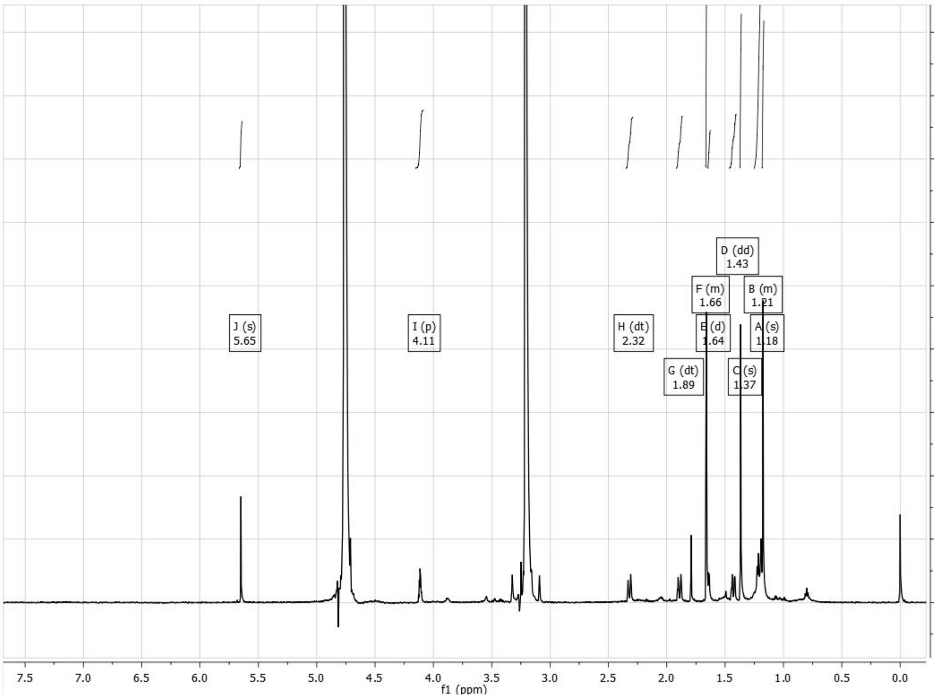

Figure S20. ^{13}C NMR spectrum of compound **6** (150 MHz, CD_3OD).

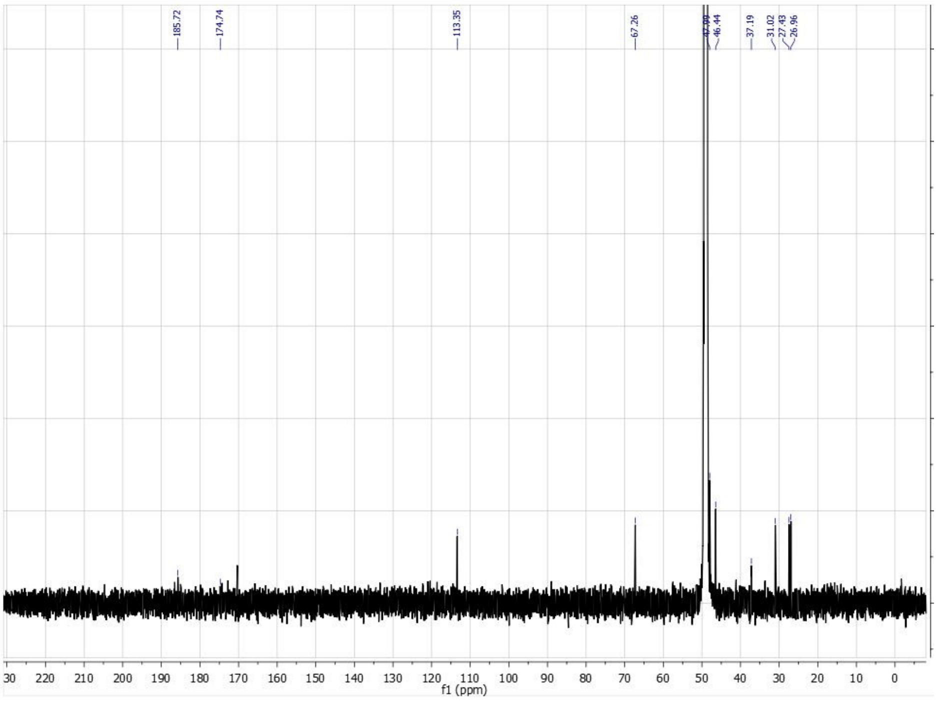

Figure S21. HR-ESIMS spectrum of compound **6**.

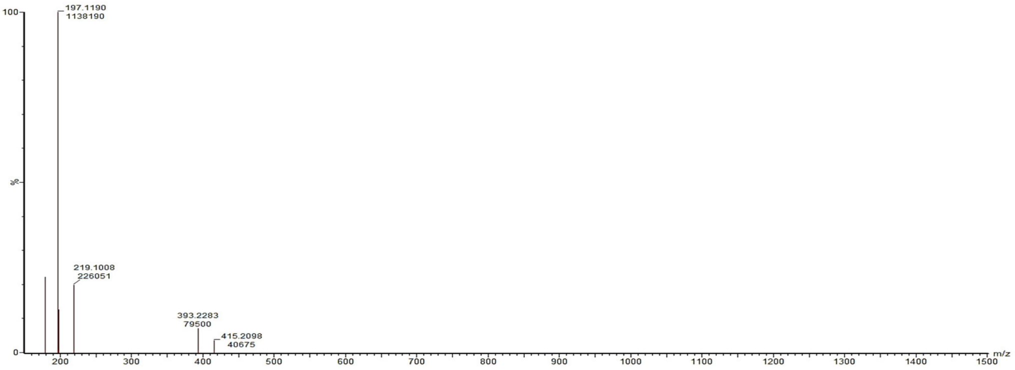

Figure S22. ^{1}H NMR spectrum of compound **7** (600 MHz, CD_3OD).

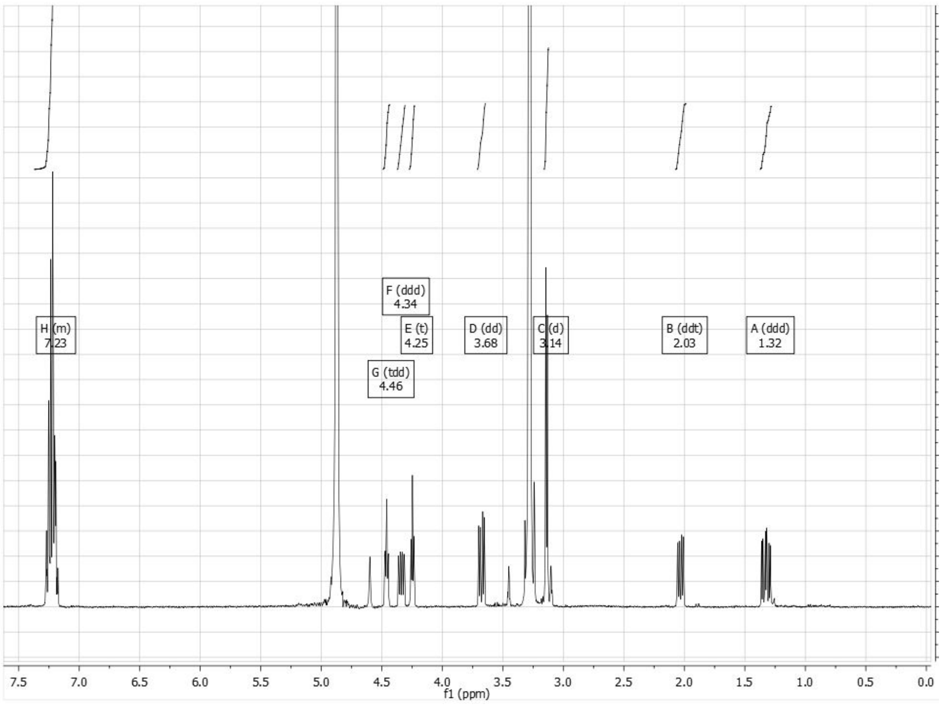

Figure S23. ^{13}C NMR spectrum of compound **7** (150 MHz, CD_3OD).

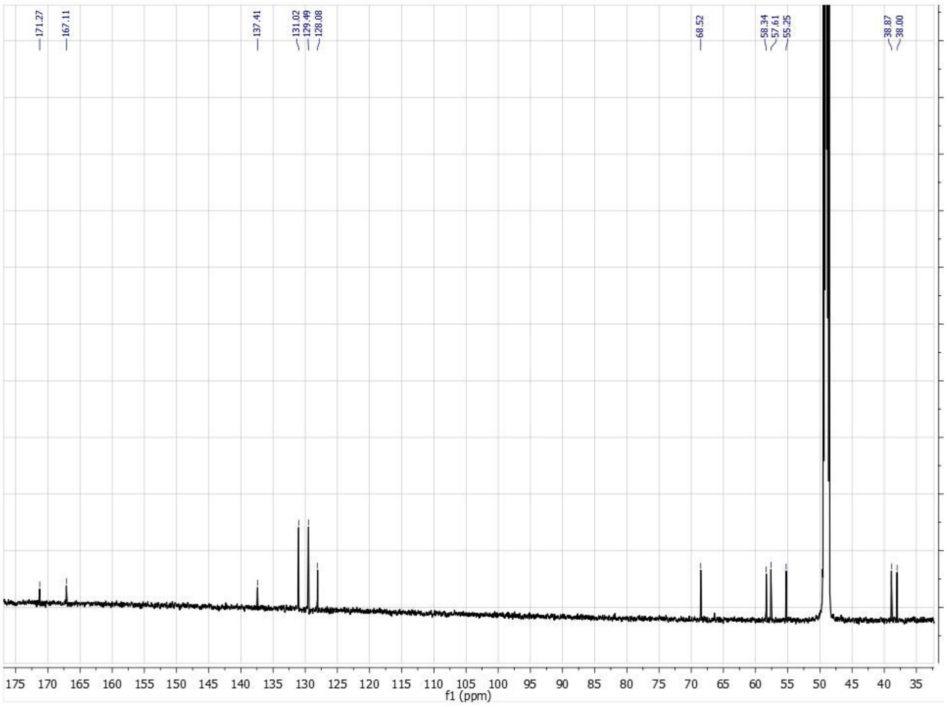

Figure S24. HR-ESIMS spectrum of compound **7**.

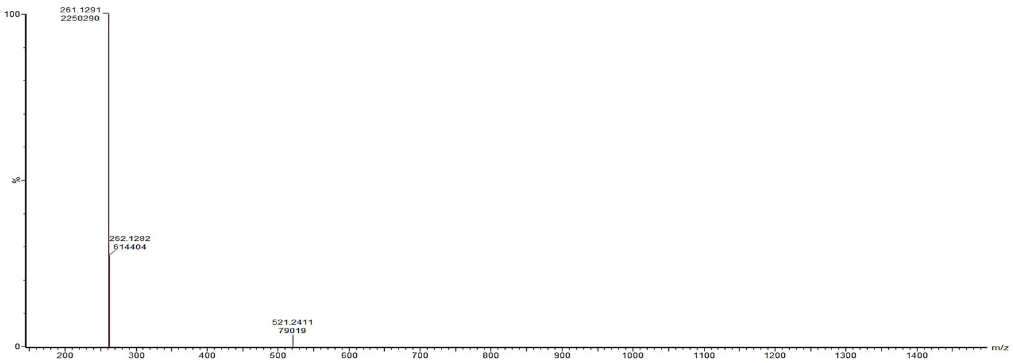

bg

Figure S25. ^{1}H NMR spectrum of compound **8** (600 MHz, DMSO-d_6).

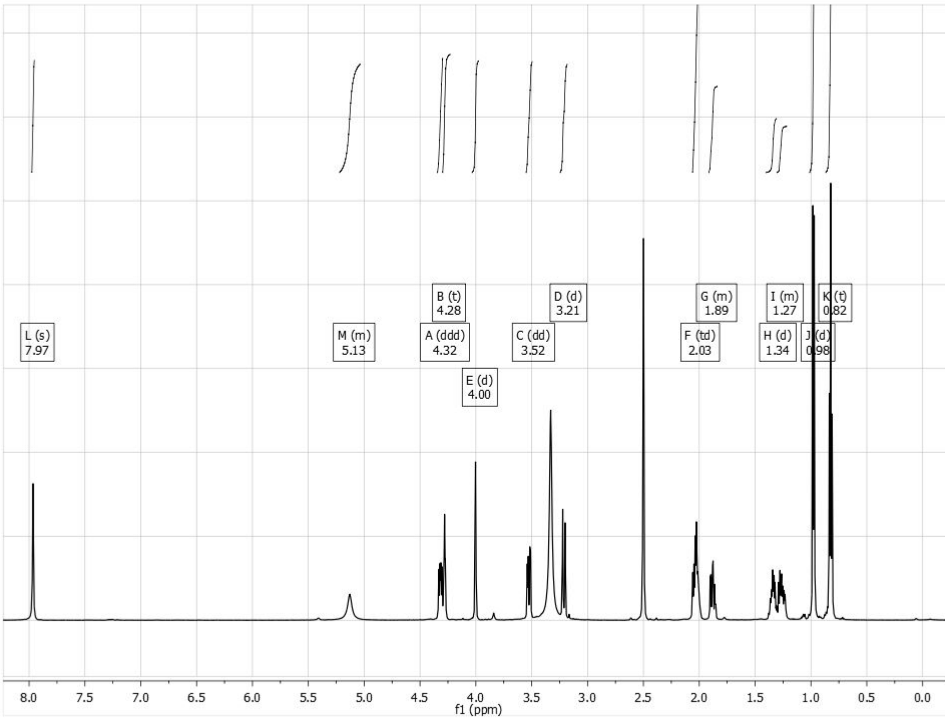

Figure S26. ^{13}C NMR spectrum of compound **8** (150 MHz, DMSO-d_6).

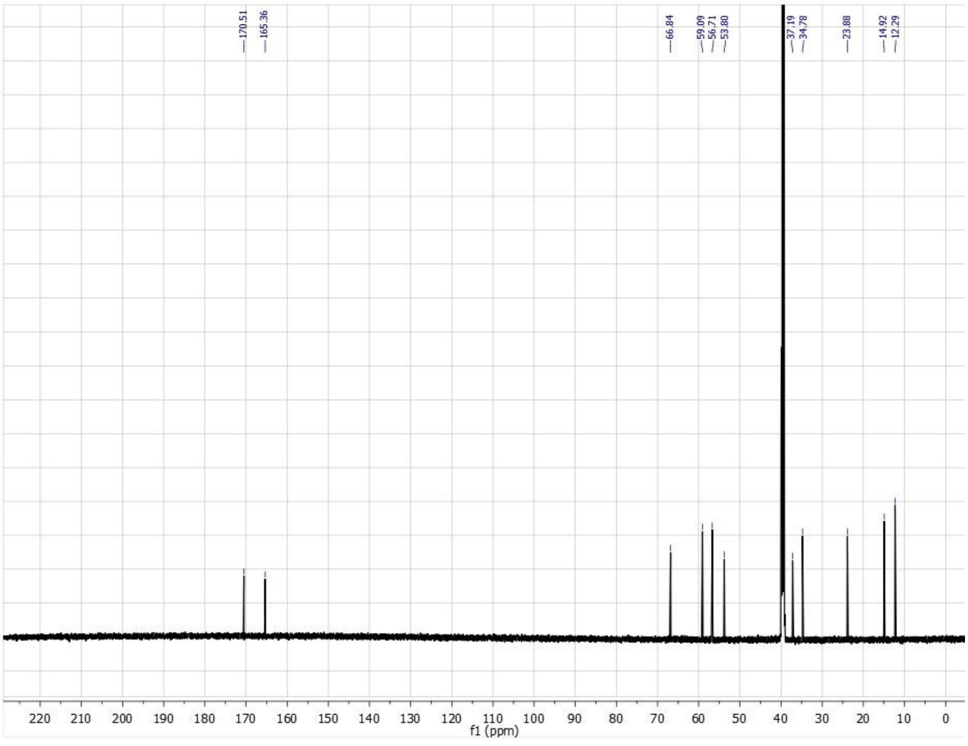

Figure S27. ^{1}H NMR spectrum of compound **8** (600 MHz, CD_3OD).

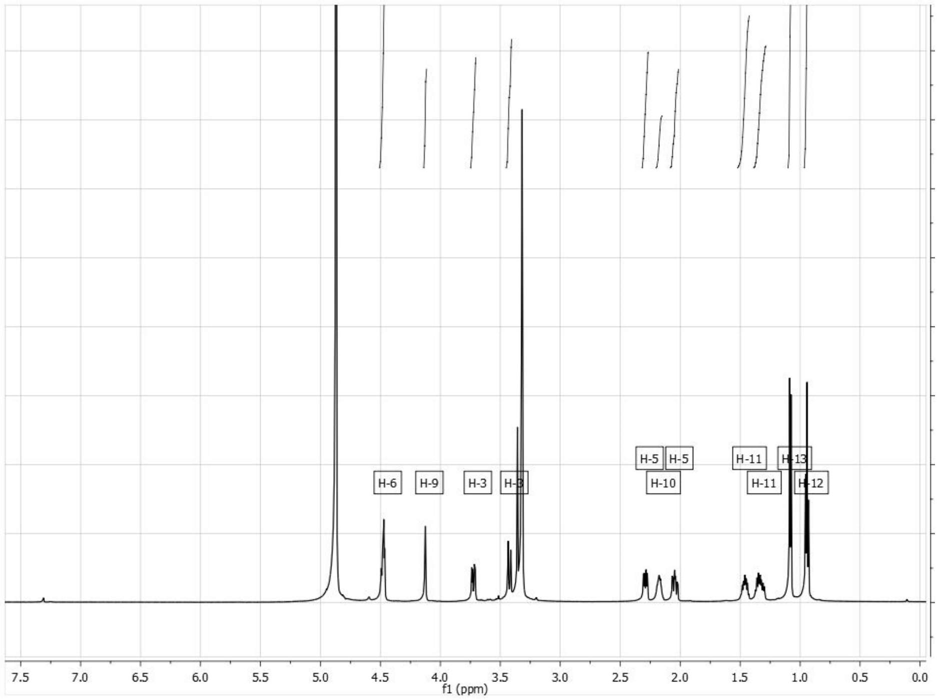

Figure S28. ^{13}C NMR spectrum of compound **8** (150 MHz, CD_3OD).

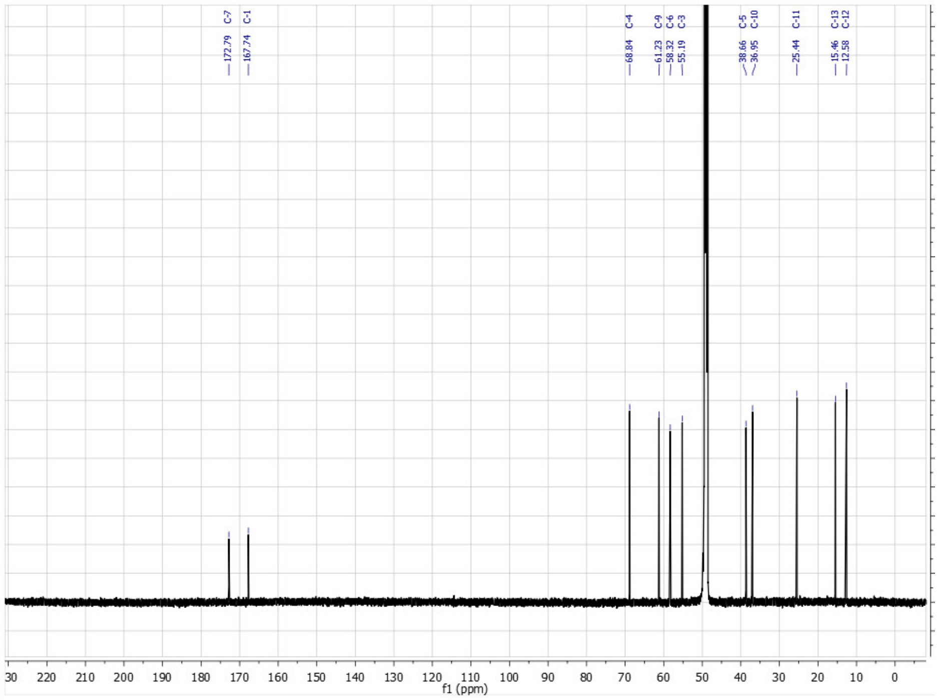

Figure S29. HSQC spectrum of compound **8** (150/600 MHz, CD_3OD).

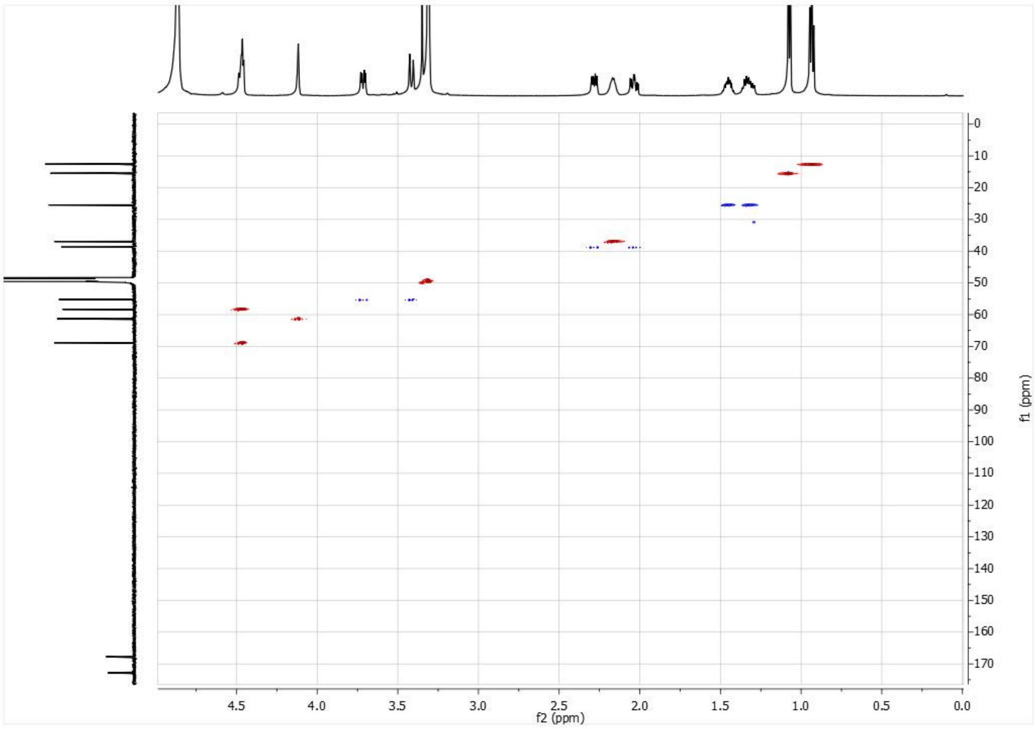

Figure S30. HMBC spectrum of compound **8** (150/600 MHz, CD_3OD).

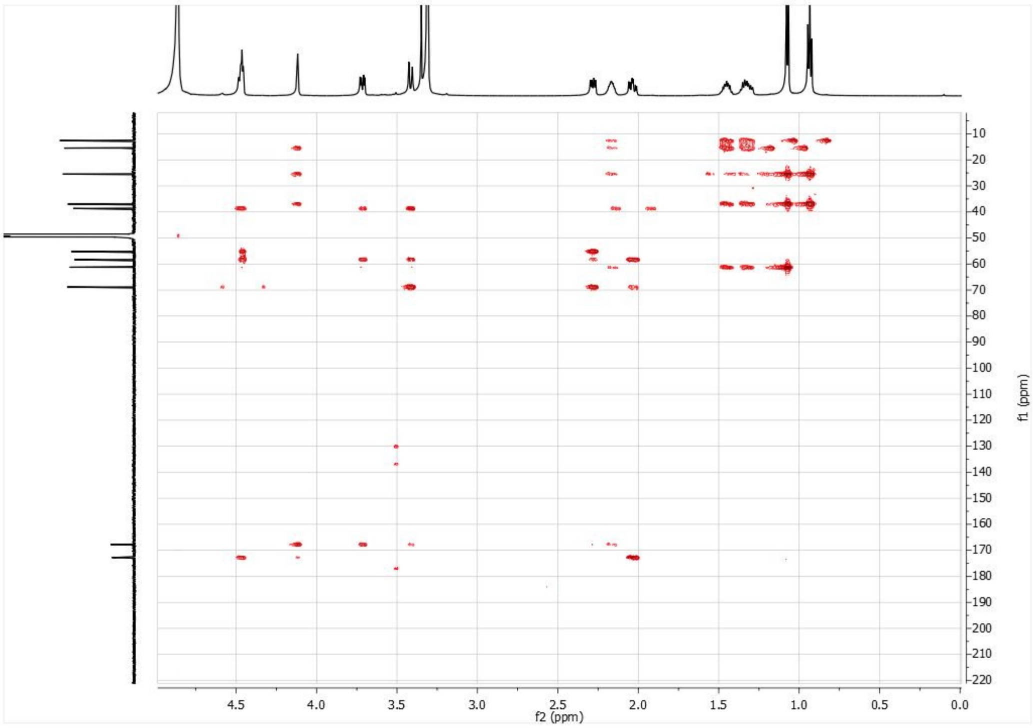

Figure S31. COSY spectrum of compound **8** (600 MHz, CD_3OD).

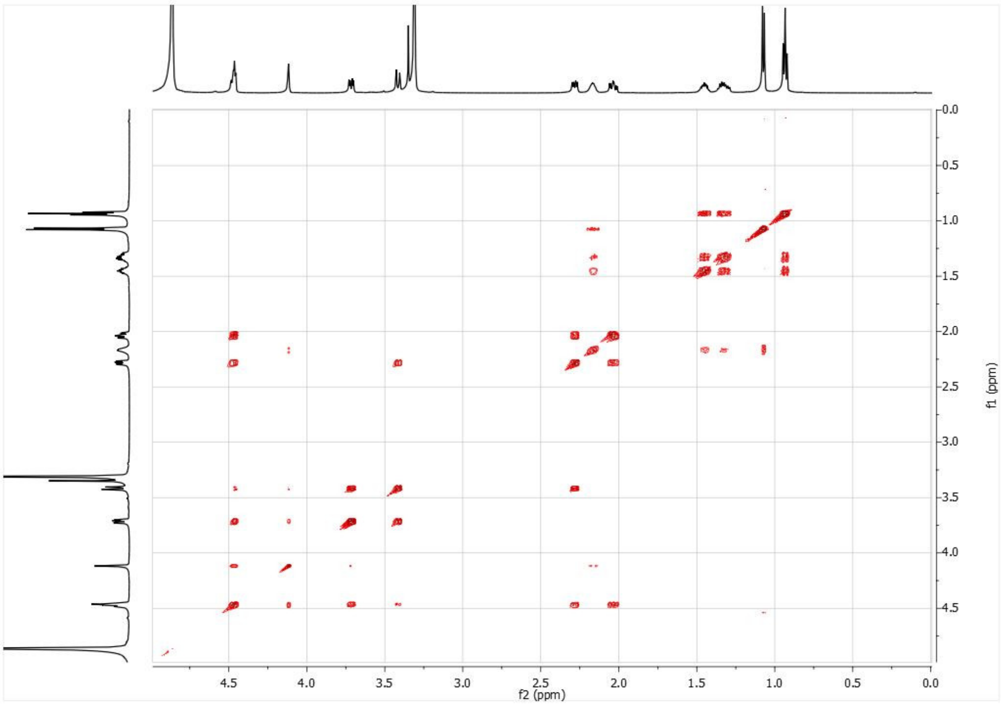

Figure S32. NOESY spectrum of compound **8** (600 MHz, CD_3OD).

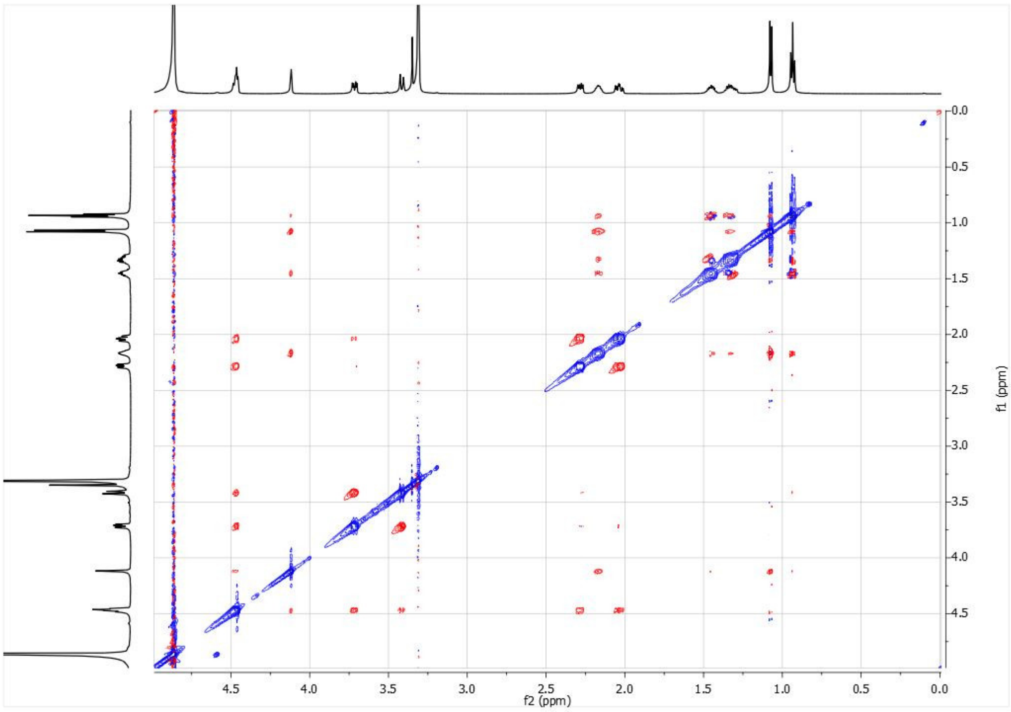

Figure S33. HR-ESIMS spectrum of compound **8**.

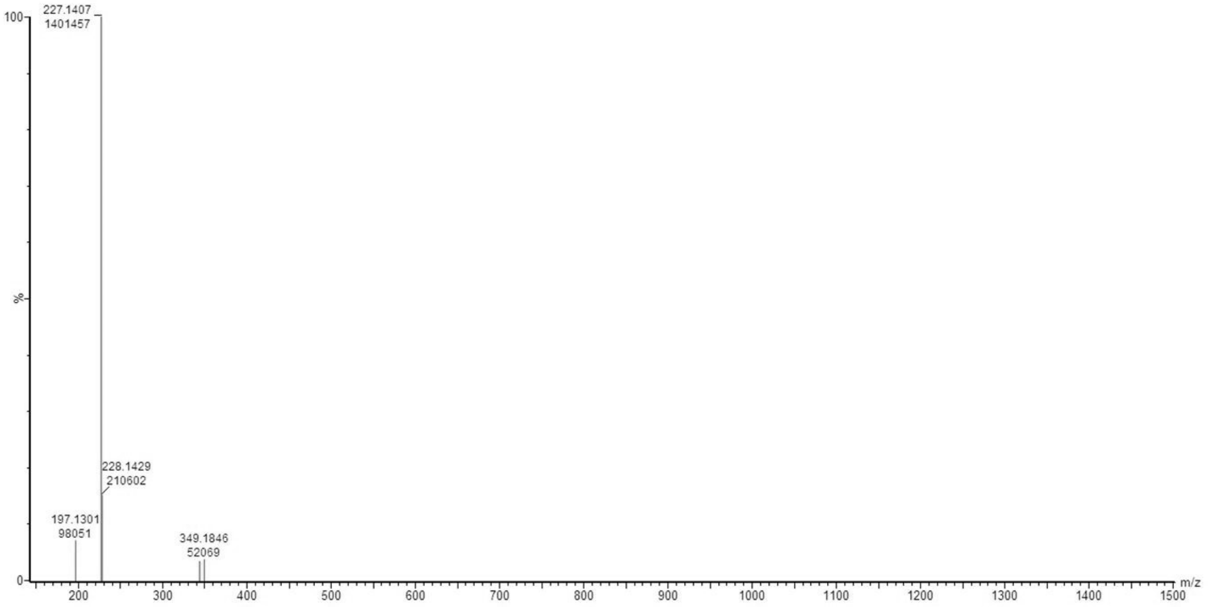

References

1. Kim, S.; Chen, J.; Cheng, T.; Gindulyte, A.; He, J.; He, S.; Li, Q.; Shoemaker, B.A.; Thiessen, P.A.; Yu, B.; Zaslavsky, L.; Zhang, J.; Bolton E.E. PubChem update: improved access to chemical data. *Nucleic Acids Res.* **2019**; *47*, 1102–1109.
2. Vorkas, P.A.; Isaac, G.; Anwar, M.A.; Davies, A.H.; Want, E.J.; Nicholson, J.K.; Holmes, E. Untargeted UPLC-MS profiling pipeline to expand tissue metabolome coverage: application to cardiovascular disease. *J. Anal. Chem.* **2015**, *87*, 4184-4193.
3. Noda, N.; Tsunefuka, S.; Tanaka, R.; Miyahara, K. Isolation of two 1-*O*-alkyl-*sn*-glycero-3-phosphocholines from the earthworm, *Pheretima asiatica*. *Chem. Pharm. Bull.* **1992**, *40*, 1349-1351.
4. Warner, H.; Lands, W. Metabolism of plasmalogen: II. The determination of alkenyl ethers in the presence of free aldehydes. *J. Lipid Res.* **1963**, *4*, 216-220.
5. Noda, N.; Tanaka, R.; Nishi, M.; Inoue, S.; Miyahara, K. Isolation and characterization of seven lyso platelet-activating factors and two lyso phosphatidylcholines from the crude drug" Suitetsu"(the leech, *Hirudo nipponica*). *Chem. Pharm. Bull.* **1993**, *41*, 1366-1368.
6. Pruzanski, W.; Lambeau, L.; Lazdunsky, M.; Cho, W.; Kopilov, J.; Kuksis, A., Differential hydrolysis of molecular species of lipoprotein phosphatidylcholine by groups IIA, V and X secretory phospholipases A_2. *BBA-Mol. Cell. Biol. L.* **2005**, *1736*, 38-50.
7. Sablina, M.A.; Ushakova, I.P.; Serebrennikova, G.A. Synthesis of analogues of platelet activating factor: 1(3)-*O*-[2′-(*R*,*S*)-methoxyhexadecyl]-2-*O*-acetyl-*sn*-glycero-3(1)-phosphocholines. *Mendeleev Commun.* **1995**, *1*, 6-7.
8. Shi, Q., Iin, Sh.; Xiang, X.; Tian, J.; Huang, R.; Li, S.; Chen, Ch.; Xu, H.; Song, Ch. The metabolic change in serum lyso-glycero-phospholipids intervened by triterpenoid saponins from kuding tea on hyperlipidemic mice. *J. Funct. Foods* **2019**, *10*, 7782-7792.
9. Qi, L.; Zhu, W.; Qin, Y.; Chen, Y. Metabolite marker for diagnosing and distinguishing coronary atherosclerosis and stable angina pectoris, **2016**, CN105445408A.
10. Riccardis, F.; Minale, L.; Iorizzi, M.; Debitus, C.; Lévi, C. Marine sterols. side-chain-oxygenated sterols, possibly of abiotic origin, from the new Caledonian sponge *Stelodoryx chlorophylla*. *J. Nat. Prod.* **1993**, *56*, 282-287.
11. Lyons, M.A.; Brown, A.J. 7-ketocholesterol. *Int. J. Biochem. Cell Biol.* **1999**, *31*, 369-375.
